# PD-L1/Lag3 Bispecific Immune Checkpoint Blocking Nanocage Exhibits Potent Antitumor Activity beyond Dual Blockade of PD-L1 and Lag3

**DOI:** 10.34133/bmr.0362

**Published:** 2026-05-07

**Authors:** Seok-Min Lee, Minseong Kim, Chanju Lee, Minah Lee, Hee Jung Yoon, Byungheon Lee, Eun Jung Park, Soyoun Kim

**Affiliations:** ^1^ Department of Biochemistry and Cell Biology, School of Medicine, Kyungpook National University, Daegu 41944, Republic of Korea.; ^2^ BK21 Plus KNU Biomedical Convergence Program, Department of Biomedical Science, School of Medicine, Kyungpook National University, Daegu 41944, Republic of Korea.; ^3^ CMRI, School of Medicine, Kyungpook National University, Daegu 41944, Republic of Korea.; ^4^ Immuno-oncology Branch, Department of Cancer Biomedical Science, Graduate School of Cancer Science and Policy, National Cancer Center, Goyang 10408, Republic of Korea.

## Abstract

The therapeutic efficacy of blocking programmed cell death protein 1 and programmed cell death ligand 1 (PD-L1) can be augmented through combination with additional immune checkpoint inhibitors. Lymphocyte-activation gene 3 (Lag3) is an inhibitory checkpoint receptor that contributes to T-cell exhaustion and tumor immune evasion. We hypothesized that cotargeting PD-L1 and Lag3 using a human ferritin nanocage platform could provide an alternative therapeutic strategy to monoclonal antibodies (mAbs) against PD-L1 or Lag3 in modulating antitumor immune responses. Here, we describe a PD-L1/Lag3 bispecific immune checkpoint-blocking ferritin nanocage (P1L2) displaying 24 PD-L1 binding peptides and 24 Lag3 binding peptides on its surface. P1L2 demonstrated simultaneous binding to both PD-L1 and Lag3 and exhibited stronger immune-stimulatory activity than either parent nanocage or mAbs against PD-L1 or Lag3. In a syngeneic mouse colon cancer model, P1L2 specifically targeted tumors and substantially suppressed tumor growth compared with each parent nanocage alone or in combination. Moreover, P1L2 showed more potent antitumor activity than a mAb against PD-L1 or Lag3 alone or in combination. These findings demonstrate that the PD-L1/Lag3 bispecific immune checkpoint-blocking ferritin nanocage is a promising nanomedicine for cancer immunotherapy and has potential applications against various solid tumors.

## Introduction

Immune checkpoint blockers (ICBs), particularly monoclonal antibodies (mAbs) targeting programmed cell death protein 1 (PD-1) and programmed cell death ligand 1 (PD-L1), have exhibited substantial therapeutic efficacy across a range of cancers [1]. There is high expression of PD-1 on activated or exhausted T cells within the tumor microenvironment (TME), while PD-L1 is expressed not only by tumor cells but also by immune cells such as tumor-associated macrophages (TAMs). The interaction between PD-1 and PD-L1 results in the inhibition of T-cell activation, and the tumor is able to evade the host immune system effectively [2]. Many mAbs targeting PD-1 or PD-L1 have been developed and clinically used in multiple cancer indications [3,4]. However, only a limited proportion of patients achieve durable responses, largely due to resistance mechanisms within the TME [5]. For example, chronic antigen exposure in the TME results in progressive dysfunction or exhaustion of tumor-infiltrating T cells. Exhausted T cells demonstrate decreased proliferative capacity, altered metabolic activity, impaired cytokine production, and persistent high expression of multiple immune checkpoint receptors, including PD-1, T cell immunoreceptor with immunoglobulin and immunoreceptor tyrosine-based inhibitory motif domains, T cell immunoglobulin and mucin domain-containing protein 3, and lymphocyte-activation gene 3 (Lag3) [6].

Recent studies suggest that combination strategies, such as coblockade of these checkpoint receptors, may be necessary to elicit a robust antitumor immune response and be therapeutically effective [7,8]. Specifically, concurrent administration of anti-Lag3 and anti-PD-1 mAbs has shown synergistic benefits compared with blocking either receptor alone [9,10]. Recently, a Lag3-blocking mAb (relatlimab) combined with a PD-1-blocking mAb (nivolumab) improved progression-free survival in melanoma patients by 2.5-fold compared with nivolumab monotherapy. This combination therapy (nivolumab plus relatlimab) has since been approved by the US Food and Drug Administration for the treatment of unresectable or metastatic melanoma, underscoring the clinical efficacy of this dual-blockade approach [11].

Lag3 is a type I transmembrane protein characterized by 4 extracellular immunoglobulin-like domains that is structurally similar to CD4 and thus also binds to major histocompatibility complex class II (MHC-II) molecules [12]. Lag3 is primarily expressed on T cells, natural killer cells, and dendritic cells (DCs), where it plays an important role in maintaining immune tolerance [13]. Although the precise mechanisms of Lag3 in these immune cells remain unclear [14], its engagement on the T-cell surface negatively regulates activation, proliferation, effector functions, and homeostasis in both CD8^+^ and CD4^+^ T cells. Several potential ligands of Lag3 have been identified, including LSECtin/CLEC4G, galectin-3, α-synuclein, and fibrinogen-like protein 1 (FGL-1) [15–17]. Notably, increased expression of Lag3 on tumor-infiltrating lymphocytes (TILs) has been observed across various solid tumor types and is correlated with adverse clinical outcomes [18]. However, clinical evaluations of mAbs targeting Lag3 have shown that anti-Lag3 monotherapy produces only modest therapeutic effects against tumors [19,20].

Small-molecule ICB peptides have gained interest as potential alternatives to cancer immunotherapy antibodies [21]. For instance, a PD-L1 binding peptide (PD-L1pep1), identified using phage display, demonstrated inhibition of PD-1/PD-L1 interactions and reinvigoration of T cells [22]. More recently, Lag3 binding peptides (Lag3pep1 and Lag3pep2) were discovered through phage library screening and demonstrated synergistic antitumor activity when combined with either a PD-L1 blocking antibody or a PD-L1 binding peptide. Despite their advantages, including deep tissue penetration, low immunogenicity, and reduced development costs, peptide drugs face limitations such as weak binding affinity, rapid renal clearance, and susceptibility to degradation [23,24]. To address these challenges, we employed a ferritin nanocage platform, which enhances peptide stability and affinity by displaying multiple copies on its surface, thereby extending half-life and improving therapeutic efficacy [25,26].

Ferritin nanocages consist of 24 monomer subunits that self-assemble into naturally occurring protein-based nanoparticles, offering superior biocompatibility, biostability, and biodegradability relative to synthetic polymers [27,28]. Their nanoscale size improves the circulation of attached ligands, while the enhanced permeability and retention (EPR) effect promotes passive tumor accumulation [29]. Additionally, the hollow inner cavity of ferritin nanocages permits encapsulation of chemotherapeutic agents, such as doxorubicin (Dox), enabling combination therapy directly in the tumor site [30,31]. Notably, human ferritin heavy-chain nanocages can traverse the blood–brain barrier (BBB) through interaction with transferrin receptor 1 (TfR1), which is highly expressed on both the endothelial cells of the BBB and glioma cells [32,33]. Given these advantages, ferritin nanocages have been widely utilized—as medical imaging contrast agents [34], targeted drug delivery systems [35,36], vaccine development [37], and diagnostic or therapeutic nanoparticle carriers [25,38].

In prior studies, we engineered a ferritin nanocage specifically targeting PD-L1 by displaying 24 PD-L1 binding peptides. This construct effectively disrupted the PD-1/PD-L1 interaction, demonstrating that ferritin nanocages provide a valuable platform for enhancing the stability and affinity of peptide ligands. Moreover, the PD-L1-targeting nanocage could encapsulate the chemotherapeutic drug Dox and exhibited potent antitumor activity through the combined effects of ICB and chemotherapy [39]. We also found that the PD-L1-targeting nanocage efficiently crossed the BBB and targeted brain tumors, resulting in significant tumor growth suppression in an orthotopic intracranial tumor model [40].

Here, we describe a PD-L1/Lag3 bispecific immune checkpoint-blocking nanocage (Bis-ICBN) that simultaneously displays 24 PD-L1 binding peptides (PD-L1pep1) and 24 Lag3 binding peptides (Lag3pep2) on its surface. The PD-L1/Lag3 Bis-ICBN is expected to inhibit tumor growth more effectively than a combination of PD-L1 and Lag3 mono-blocking nanocages, while potentially minimizing side effects. As proof of principle, we evaluated the antitumor efficacy of PD-L1/Lag3 Bis-ICBN in mouse colon cancer models, demonstrating its superior activity and highlighting its applicability as a novel approach to address the limitations of PD-L1-targeted immune checkpoint blockade therapy.

## Materials and Methods

### Generation of Lag3pep-ferritin nanocages and PD-L1/Lag3-ferritin nanocages

The recombinant plasmid for expressing Lag3pep-ferritin nanocages was constructed using a previously described modified pET28 vector (Novagen) [41]. Oligonucleotides encoding Lag3 binding peptide 1 (Lag3pep1, CIRNDPAVC) or Lag3 binding peptide 2 (Lag3pep2, CSVLNASGC) were inserted between the KpnI and NheI sites of a short form of the ferritin heavy chain (sFTH, residues 1 to 161) to produce N-terminal ligated Lag3pep1-N-sFtH (N1) or Lag3pep2-N-sFtH (N2), respectively, as previously described [26]. The Lag3pep1 or Lag3pep2 encoding oligonucleotides were inserted between the BamHI and ApaI sites of the ferritin heavy chain (FTH, residues 1 to 183) to produce Lag3pep1-L-FtH (L1) or Lag3pep2-L-FtH (L2), respectively, consistent with prior reports [25]. Additionally, we inserted Lag3pep1 or Lag3pep2 oligonucleotides between SpeI and XhoI sites of the sFTH to yield C-terminal fusion constructs Lag3pep1-C-sFtH (C1) or Lag3pep2-C-sFtH (C2), respectively [25].

To engineer bispecific PD-L1/Lag3-ferritin nanocages, oligonucleotides encoding the PD-L1 binding peptide (PD-L1pep1, CLQKTPKQC) were fused to the N-terminus of ferritin monomer, while Lag3pep1 or Lag3pep2 was inserted into the loop between helices IV and V, resulting in the construction of P1L1 and P1L2 ferritin nanocages, respectively.

### Protein purification and 3D structure modeling

We overexpressed proteins in *Escherichia coli* strain BL21 (DE3) cells and purified them following the protocol described previously [41]. In brief, we induced protein expression with 0.5 mM isopropyl β-D-1-thiogalactopyranoside at 37 °C for 4 h at an optimal cell density at 600 nm of 0.5. We purified the expressed proteins from cell lysates using Ni-NTA agarose beads (Thermo Fisher Scientific, MA, USA). The beads were washed with buffer containing 0.1% Triton X-114 (20 mM Tris, pH 7.4; 500 mM NaCl; 30 mM imidazole; and 0.5 mM dithiothreitol [DTT]) to remove endotoxins [42]. The beads were then thoroughly rinsed to remove the detergent, after which the proteins were eluted stepwise with Tris-based saline (TBS) buffer (20 mM Tris, pH 8.0; 150 mM NaCl; and 2 mM DTT) containing 100 or 300 mM imidazole, and then dialyzed against TBS buffer to remove imidazole. For in vivo experiments, apo-nanocages were generated by adding 10 mM ethylenediaminetetraacetic acid. We verified protein purity and homogeneity via sodium dodecyl sulfate–polyacrylamide gel electrophoresis. For fluorescence labeling, purified proteins were conjugated with Flamma 675 or Flamma 488 dyes (BioActs, Korea) following the manufacturer’s instructions and used for in vivo targeting or cellular binding analyses, respectively.

We modeled the structure of the PD-L1/Lag3pep-ferritin subunit using MODELLER v9.12 described previously [39]. We generated and assembled monomeric structures into a PD-L1/Lag3-ferritin nanocage using PyMOL. The 20 structures with the fewest violations were further refined, and the lowest energy structure was chosen as the final model using AMBER.

### Physical characterization of PD-L1/Lag3-ferritin nanocages

The sizes of PD-L1/Lag3-ferritin nanocages (P1L1 and P1L2) were characterized utilizing transmission electron microscopy (TEM), dynamic light scattering (DLS), and zeta potentials following the previous description [41]. For TEM examination, protein samples at a concentration of 0.5 mg/ml were deposited as a single droplet onto copper grids coated with a carbon film and subsequently subjected to negative staining with uranyl acetate solution. Imaging was performed using a FEI Tecnai electron microscope located at the Core Laboratory facility of Kyungpook National University.

### Cell cultures

We obtained cell lines and reagents from the American Type Culture Collection (Manassas, VA). We maintained human embryonic kidney (HEK) 293T cells, human breast carcinoma cells (MDA-MB-231), mouse glioma cells (CT-2A and GL26), and murine colon adenocarcinoma cells (MC38, C57BL/6 origin) in Dulbecco’s Modified Eagle’s Medium. Human acute T-cell leukemia cells (Jurkat T) and human acute monocytic leukemia cells (THP-1) were maintained in RPMI-1640 medium. All culture media were supplemented with 10% fetal bovine serum (Thermo Fisher Scientific), 100 U/ml penicillin, and 100 μg/ml streptomycin. Cells were cultured at 37 °C in a humidified atmosphere with 5% CO_2_.

### Immunofluorescence and flow cytometry analyses of cellular binding

For immunofluorescence analysis, we used HEK 293T cells (1.25 × 10^5^), transfected with Lag3 plasmid DNA. In order to minimize nonspecific binding, cells were seeded on 4-well chamber slides and incubated with 1% bovine serum albumin (BSA) at room temperature (RT) for 1 h. Following this blocking step, the cells were incubated at 4 °C for 1 h with 100 nM of Lag3pep-ferritin nanocages (N1, L1, C1, N2, L2, or C2) or PD-L1/Lag3pep-ferritin nanocages (P1L1 or P1L2), with wild-type ferritin heavy chain (wFTH) serving as a control. Subsequently, the cells were treated with an anti-ferritin primary antibody (Abcam, ab65080) followed by an anti-rabbit Alexa Fluor 594-conjugated secondary antibody (A11012, Invitrogen). Expression of Lag3 was evaluated using an anti-human monoclonal Lag3 antibody (Santa Cruz Biotechnology, sc-514993) at a 1:100 dilution. After antibody incubation, we fix the cells with 4% paraformaldehyde (PFA), counterstained with 4′,6-diamidino-2-phenylindole (Sigma-Aldrich), mounted using ProLong antifade reagent (Thermo Fisher Scientific), and imaged via a K1-Fluo confocal fluorescence laser scanning microscopy (Nanoscope Systems, Korea). In a separate set of experiments, we seeded MDA-MB-231 cells (1 × 10^5^) onto 8-well chamber slides and cultured them overnight. These cells were then incubated at 4 °C for 1 h with 100 nM PD-L1pep1-ferritin nanocages (P1) or PD-L1/Lag3pep-ferritin nanocages (P1L1 or P1L2). We then treated the cells with the anti-ferritin antibody (Abcam, ab65080) and an anti-rabbit Alexa Fluor 488-conjugated secondary antibody (Invitrogen, A11008) for subsequent immunofluorescence analysis [39].

For flow cytometry analyses, Jurkat T cells (2.5 × 10^5^) were plated in 12-well culture plates and stimulated with phorbol 12-myristate 13-acetate (Sigma-Aldrich) at 50 ng/ml, ionomycin (Sigma-Aldrich) at 1 μg/ml, and chloroquine (Abcam) at 100 μM for 48 h. Following stimulation, cells (1 × 10^6^) were resuspended in culture medium supplemented with 1% BSA and incubated at RT for 1 h. The cells were then incubated with 100 nM fluorescent labeled Lag3pep-ferritin nanocages (designated as N1, L1, C1, N2, L2, or C2) at 4 °C for 1 h. To ensure uniform total protein concentration across all samples and facilitate direct comparison of fluorescence binding, unlabeled protein was added as a normalization control. Alternatively, bound ferritin constructs were measured by anti-ferritin antibody (Abcam, ab75973). After washing steps, the cells were analyzed using an Attune NxT flow cytometer (Thermo Fisher Scientific), and the resulting data were processed with the instrument’s proprietary software.

### Surface plasmon resonance analysis

The interactions of Lag3pep-ferritin nanocages (N1, L1, C1, N2, L2, C2, P1L1, and P1L2) were investigated at 25 °C utilizing a surface plasmon resonance (SPR) spectrometer (SR7500 DC, Reichert Inc., NY, USA). Human Lag3-Fc fusion protein (Acro Biosystems, LA3-H5255) was immobilized onto Protein A sensor chips (~1,000 resonance units). To determine the binding affinities of Lag3pep-ferritin nanocages, varying concentrations of ferritin nanocages were flowed over the immobilized Lag3-coated surface at a flow rate of 45 μl/min. Following each association and dissociation, the surface was regenerated by injecting 25% ethylene glycol and 1 M NaCl for 30 s. Resonance unit data were collected and analyzed using Scrubber 2.0 (BioLogic Software, Australia). Equilibrium dissociation constants (*K*_D_) were calculated by fitting steady-state binding data to a 1:1 binding model using GraphPad Prism 3.0 [22]. Interaction of P1L2 with PD-L1 was analyzed as previously described [39].

### Cell-based blocking assay

THP-1 cells expressing human leukocyte antigen-DR isotype (HLA-DR, a subtype of human MHC-II) were used to assess whether Lag3-targeting ferritin nanocages could block interaction between Lag3 and HLA-DR. THP-1 cells were stimulated with 50 ng/ml interferon-gamma (IFN-γ) (PeproTech) for 48 h and then incubated with 400 ng of hLag3-Fc protein (18.3 nM; Acro Biosystems) in the presence or absence of Lag3pep-ferritin nanocages (L1, L2, P1L1, or P1L2; 183 nM) at 4 °C for 30 min. As a positive control, the same molar concentration (183 nM) of anti-human Lag3 blocking antibody (AdipoGen Life Sciences) was used. Following incubation, the mixture was applied to 5 × 10^5^ THP-1 cells and further incubated at 4 °C for an additional 30 min. Cell-bound hLag3-Fc protein was stained using an anti-Fc-PE antibody (eBioscience). After washing, we analyzed the cells through an Attune NxT flow cytometer (Thermo Fisher Scientific).

### Animals

We performed all animal experiments with institutional guidelines and approved by the Institutional Animal Care and Use Committee of Kyungpook National University (Approval No. KNU 2024-0546). We took care to do everything possible to minimize animal suffering. We purchased female C57BL/6 wild-type mice (6 weeks old) from Orient Bio Inc. (Seongnam, South Korea) and maintained in a pathogen-free animal facility.

### Coculture of CD8^+^ T cells with tumor cells for analysis of T-cell activity

C57BL/6 wild-type mice were administered MC38 cells (1 × 10^6^) to enhance immunogenicity. After 1 week, we collected spleens from tumor-bearing mice, and isolated CD8^+^ T cells utilizing a CD8^+^ T-cell isolation kit (Stem Cell Technologies). For activation, CD8^+^ T cells (1 × 10^6^) were incubated with 25 μl of Nanobeads Mouse T-Activator (Thermo Fisher Scientific, 11456D) in the presence of mouse IL-2 and IL-15 (R&D Systems) for 48 h.

Subsequently, we resuspended the activated CD8^+^ T cells in complete RPMI medium and cocultured the cells with MC38 cells at a 10:1 ratio. The cocultures were then treated with either anti-mouse Lag3 blocking antibody (clone BE0174, Bio X Cell) or anti-mouse PD-L1 blocking antibody (clone 10F.9G2, Bio X Cell) at a concentration of 10 μg/ml (67 nM), ferritin constructs (P1, L2, P1L2, or wFTH) at 50 nM, or left untreated, followed by incubation for 24 h.

Concentrations of IFN-γ and Granzyme B were measured from the supernatants by performing enzyme-linked immunosorbent assay (ELISA) using the IFN-γ ELISA kit (BioLegend, 430804) and the Granzyme B ELISA kit (Thermo Fisher Scientific, 88-8022-88), respectively, following the manufacturers’ protocols. For proliferation assays, T cells were labeled with 5 μM carboxyfluorescein succinimidyl ester (Thermo Fisher Scientific) at 37 °C for 20 min prior to coculture. After 24 h, T cells were harvested, and CD3^+^ T cells were gated by flow cytometry to assess cellular proliferation. Cytotoxicity against tumor cells was evaluated using a lactate dehydrogenase (LDH) assay (CytoTox96 Nonradioactive Cytotoxicity Assay, Promega, G1780), according to the manufacturer’s instructions. For quantitative measurement of viable MC38 cells remaining after CD8^+^ T cell-mediated cytotoxicity, MC38 tumor cells and CD8^+^ T cells were labeled with CellTrace Far Red (Invitrogen, C34572) and CellTrace Green (Invitrogen, C34554) per the manufacturer’s instructions, washed to remove excess dye, and then cocultured for 24 h under the indicated conditions. After coculture, Far-Red fluorescence of the remaining MC38 cells was measured using a FlexStation 3 microplate reader (Molecular Devices, USA).

For functional assessment of T-cell responses against glioma cells, IFN-γ production and degranulation were evaluated following coculture of CD8^+^ T cells with GL26 and CT2A tumor cells in the presence of glial cells, as previously described [40]. Briefly, primary glial cells (1 × 10^4^ cells/well) were plated in 96-well plates, and glioma cells (1 × 10^4^ cells/well) were added 3 h prior to coculture. CD8^+^ T cells were obtained from the spleen and lymph nodes of wild-type C57BL/6 mice using an immunomagnetic negative selection kit (STEMCELL Technologies, 19853). Prior to coculture with tumor and glial cells, T cells were preactivated for 1 h using anti-CD3/CD28 antibodies. Experimental treatments comprised wFTH, P1, L2, a combination of P1 and L2 (P1 + L2), P1L2 (0.6 μM), or vehicle buffer (equal volume). After 48 h of coculture, IFN-γ concentrations in the supernatants were quantified utilizing a mouse IFN-γ ELISA kit (R&D Systems, SMIF00). Concurrently, CD8^+^ T-cell degranulation was assessed by flow cytometry employing the BD FACS Lyric system, with data analysis conducted using FlowJo software.

### In vivo tumor targeting and biodistribution of PD-L1/Lag3 Bis-ICBN

To establish the tumor allograft model, we injected MC38 cells (1 × 10^6^ cells per mouse) subcutaneously into the flank of mice. Subsequently, Flamma 675-labeled P1L2 Bis-ICBN (~10 mg/kg), PD-L1 peptide ferritin nanocage (P1), Lag3 peptide ferritin nanocage (L2), or wFTH was administered intravenously via the tail vein (*n* = 3 mice per group). Protein dosages were calibrated to ensure equivalent fluorescence levels across treatments, facilitating direct comparison of tumor-targeting efficacy. Fluorescence imaging was performed at 1, 4, 6, 8, 12, 24, and 48 h postinjection using the IVIS Lumina imaging system (Caliper, USA), with animals maintained under continuous isoflurane anesthesia during imaging sessions. At 48 h post-administration, tumors and major organs were excised for ex vivo analysis using the IVIS Lumina system. Quantitative assessment of fluorescence intensity within tumors was conducted by measuring total photon emission per square centimeter per steradian (p/s/cm^2^/sr) within defined regions of interest, utilizing Living Image Software (Caliper, USA).

### In vivo antitumor therapy with PD-L1/Lag3 Bis-ICBN

An in vivo tumor model was developed by subcutaneous inoculation of MC38 (1 × 10^6^) tumor cells into the dorsal flank of 6-week-old female C57BL/6 mice. Tumor-bearing mice were randomly allocated into experimental groups to assess the antitumor efficacy of the PD-L1/Lag3 Bis-ICBN (P1L2) compared with mono-targeting ferritin nanocages, PD-L1pep-ferritin nanocage (P1), and Lag3pep-ferritin nanocage (L2), as well as the combination of P1 and L2. wFTH served as a control. Upon tumors reaching an approximate volume of 100 mm^3^, ferritin-based constructs were administered intravenously at a dose of 10 mg/kg, 3 times weekly, for a total of 5 injections (*n* = 5 per group). To compare antitumor efficacy with mAbs, mice received either single or combined treatments of anti-mouse PD-L1 blocking antibody (clone 10F.9G2, Bio X Cell) and anti-mouse Lag3 blocking antibody (clone BE0174, Bio X Cell) via intraperitoneal injection at 10 mg/kg, twice weekly for 3 total injections (*n* = 5 per group). Tumor progression was monitored for ulceration, and tumor volumes were assessed every other day using the formula: Volume = (length × width × width)/2. Concurrently, body weights were recorded bi-daily. At the end of the experiment regimen, blood and serum samples were collected for hematological analysis and for assessment of liver and kidney function, conducted at the Daegu Gyeongbuk Medical Innovation Foundation (DGMIF, Korea).

### Histological analysis

Mouse tissues were fixed in 4% PFA for at least 24 h and embedded in paraffin by the Bio-Medical Research Institute (BMRI), Kyungpook National University Hospital. Paraffin-embedded tissues were sectioned at 4 μm thickness, deparaffinized, and rehydrated. For histological evaluation, sections were stained with hematoxylin and eosin (H&E) according to standard procedures, and images were acquired at 200× magnification using a K1-Fluo Confocal Microscope (Nanoscope Systems, Daejeon, Korea). For detection of cell death, adjacent sections were subjected to Terminal deoxynucleotidyl transferase dUTP Nick End Labeling (TUNEL) staining using a TUNEL Assay Kit—BrdU-Red (Abcam, Cat. No. ab66110) according to the manufacturer’s instructions. Briefly, the sections were subjected to antigen retrieval, labeled with Br-dUTP for 1 h at 37°C, and incubated with anti-BrdU-Red antibody for 30 min at RT. The sections were then washed and mounted with ProLong Gold (Thermo Fisher Scientific). Fluorescence images were acquired at 400× magnification using the same microscope.

### Flow cytometry analysis of immune cell population in tumor tissue

At the end of the treatment period, mice were euthanized, and tumors were excised, weighed, and sectioned into approximately 2-mm^3^ fragments. Tumor tissues were then incubated in a digestion buffer containing collagenase D (5 mg/ml) prepared in RPMI-1640 medium at 37 °C for 30 min, following the protocol described previously [39]. Cells obtained from 250 μl of the resulting suspension were stained with specific antibodies at 4 °C for 30 min. Initial staining was performed using an anti-mouse CD45 antibody (BioLegend), followed by staining with anti-mouse CD3, CD4, CD8, and FoxP3 antibodies (BioLegend). Subsequent to staining, immune cell populations were quantified via flow cytometry (Thermo Fisher Scientific).

### Statistical analysis

We assessed statistical significance using analysis of variance (ANOVA) (1-way or 2-way) followed by Dunnett’s multiple comparison test for analyses involving 3 or more groups. Data are expressed as mean ± standard error (SE) derived from a minimum of 3 independent experiments. Statistical significance for in vitro binding assays and biodistribution studies was assessed using the Student *t* test.

## Results

### Design and construction of Lag3pep-ferritin nanocages

**Fig. 1.**
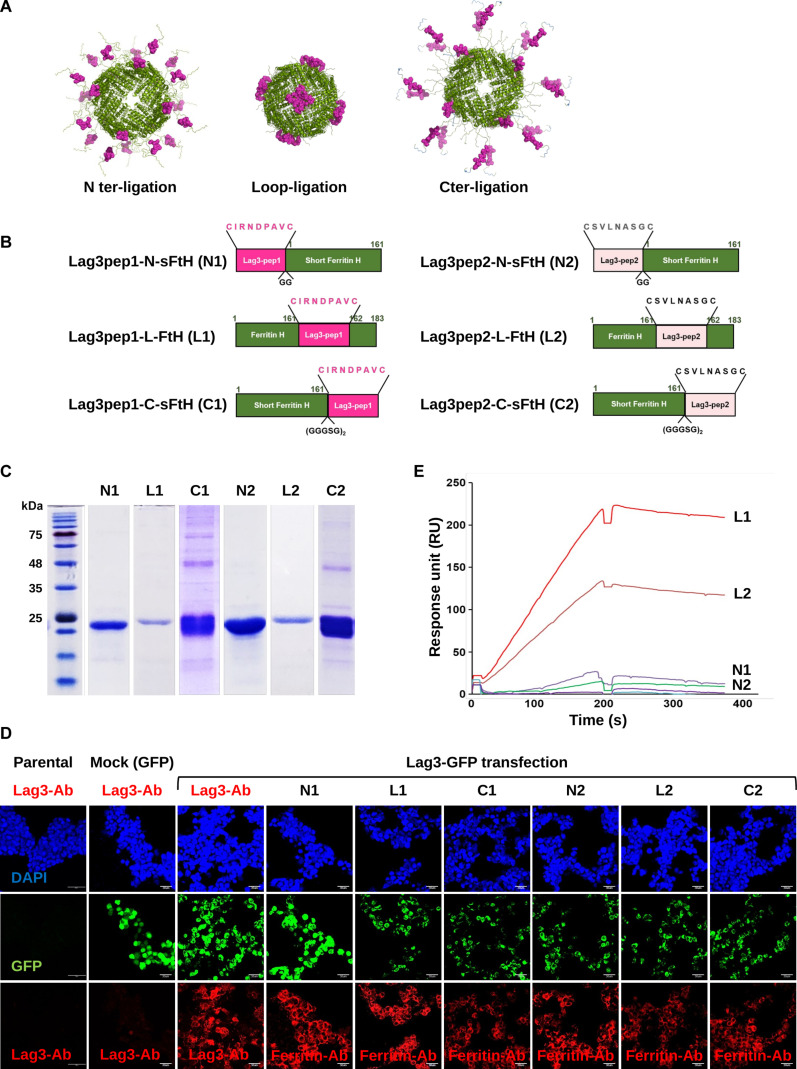
(A) Three-dimensional (3D) models of the 3 types of peptide-displaying ferritin nanocages. (B) Schematic representation of Lag3pep-ferritin nanocages. Lag3pep1 (CIRNDPAVC) or Lag3pep2 (CSVLNASGC) was fused to the N-terminus (N1 and N2), the C-terminus (C1 and C2), or the loop region of ferritin (L1 and L2). (C) Sodium dodecyl sulfate–polyacrylamide gel electrophoresis (SDS-PAGE) analysis of the purified Lag3pep-ferritin nanocages. (D) Human embryonic kidney (HEK) 293T cells expressing lymphocyte-activation gene 3 (Lag3) were incubated with Lag3pep-ferritin nanocages or wild-type ferritin heavy chain (wFTH) at 4 °C for 1 h. Binding was detected using an anti-ferritin antibody (red), nuclei were counterstained with 4′,6-diamidino-2-phenylindole (DAPI) (blue), and green fluorescent protein (GFP)-tagged Lag3 expression is shown in green. Scale bars: 30 μm. (E) Surface plasmon resonance (SPR) analysis showing the binding affinity of Lag3pep-ferritin nanocages to Lag3. Resonance units (RU) at 500 nM of each construct are shown, depicting association and dissociation kinetics.

Lag3pep1 (CIRNDAVC) and Lag3pep2 (CSVLNASGC) were identified through screening a phage-displayed peptide library. These peptides demonstrated specific binding affinity to Lag3 and, when administered in combination with a PD-L1 blocking antibody, effectively inhibited tumor growth. The FTH monomers (residues 1 to 183), consisting of 5 helical bundles, is capable of self-assembling into a 24-subunit cage-like structure. By genetically fusing peptides to 1 of 3 distinct sites—the N-terminus, the loop region between the helix IV and V, or the C-terminus of the sFTH—it is possible to display 24 copies of the peptides on the surface of the assembled ferritin cage [26,39]. The presence of multiple peptide copies synergistically enhances binding avidity; however, the pattern of peptide display varies according to the site of fusion. For example, peptides ligated at the N-terminus exhibit a dispersed distribution, whereas those ligated within the loop region tend to form clusters. C-terminal ligated peptides also exhibit a dispersed pattern, albeit less widely than N-terminal counterparts (Fig. 1A).

Correspondingly, binding kinetics differ according to the ligation site. Building on this principle, 6 ferritin constructs targeting Lag3 were designed and constructed, displaying either Lag3pep1 or Lag3pep2. Specifically, Lag3pep1 was fused to the N-terminus (N1) or C-terminus (C1) of sFTH, or inserted into the loop region (L1) of FTH. Similarly, Lag3pep2 was fused to the N-terminus (N2) or C-terminus (C2) of sFTH, or incorporated into the loop (L2) of FTH (Fig. 1B). All 6 Lag3pep-displaying ferritin constructs were expressed in *E. coli* and purified to a purity exceeding 98% (Fig. 1C). Notably, the yields of the loop-ligated constructs (L1 and L2) were lower relative to other sFTH-based constructs, which is likely attributable to the higher expression levels of sFTH in *E. coli*.

To evaluate their binding capabilities, HEK 293T cells transiently expressing green fluorescent protein (GFP)-tagged Lag3 were incubated with each ferritin construct. All 6 constructs bound Lag3 on the cell surface, and Lag3-GFP expression was confirmed via specific binding of anti-Lag3 antibodies (Fig. 1D). We next performed flow cytometry analysis using Jurkat T cells with stimulated Lag3 expression to quantitatively compare the binding of Lag3pep-displaying nanocages to immune cells (Fig. S1). The loop-ligated constructs (L1 and L2) and the N-terminally ligated N1 showed significant binding to activated Jurkat T cells when treated with fluorescence-labeled ferritin constructs. SPR measurements with purified Lag3 receptor further demonstrated that the loop-ligated constructs (L1 and L2) exhibited markedly higher affinity for Lag3 than the other constructs, indicating that peptides linked to the loop between helices IV and V efficiently bind Lag3 (Fig. 1E). Candidate selection was ultimately based on SPR analysis, which provided precise binding measurements to purified Lag3. DLS analysis of the loop-ligated ferritin constructs (L1 and L2) indicated that both L1 and L2 formed nano-sized cages with the similar mean diameters of the wFTH (Fig. S2). Based on these results, the loop-ligated constructs (L1 and L2) were selected for the development of PD-L1/Lag3 bispecific ferritin nanocages.

### Design, construction, and physical characterization of the PD-L1/Lag3 bispecific ferritin nanocages

**Fig. 2.**
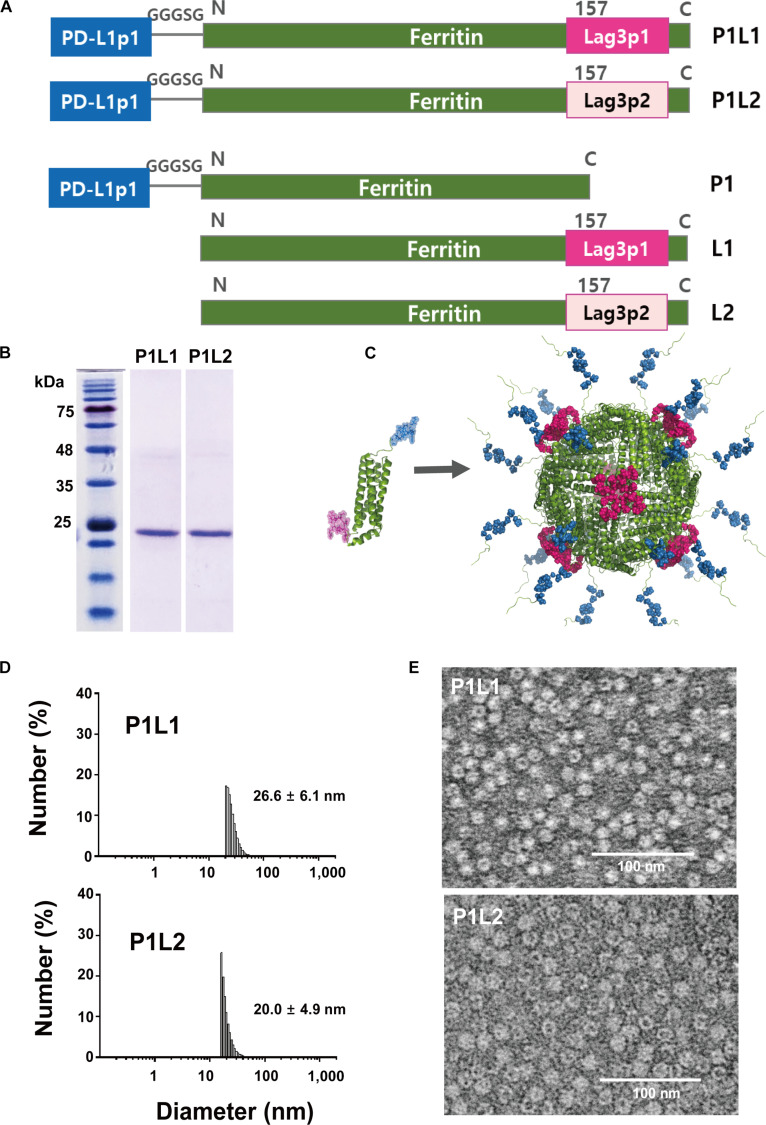
Construction and characterization of programmed cell death ligand 1 (PD-L1)/Lag3 bispecific ferritin nanocages. (A) Schematic representation of PD-L1/Lag3 bispecific ferritin nanocages (P1L1 and P1L2) and their parental nanocages (P1, L1, and L2). (B) SDS-PAGE analysis of purified PD-L1/Lag3 bispecific ferritin nanocages. (C) Three-dimensional model of the PD-L1/Lag3 bispecific ferritin nanocage generated by computational simulation. (D) Dynamic light scattering (DLS) analysis showing the size distribution of PD-L1/Lag3 bispecific ferritin nanocages. (E) Transmission electron microscopy (TEM) images confirming the cage structure and uniform size of the PD-L1/Lag3 bispecific ferritin nanocages.

In a previous study, we developed PD-L1pep-displaying ferritin nanocages, in which the PD-L1 binding peptide (PD-L1pep1, CLQKTPKQC) was fused to the N-terminus of the FTH monomer [39]. Bispecific ferritin nanocages have also been shown to retain the individual functionality of 2 different peptides when simultaneously conjugated to the N-terminus and the loop between helices IV and V, exhibiting high receptor affinity without mutual interference [26]. Based on this strategy, we designed bispecific ferritin nanocages targeting PD-L1 and Lag3. PD-L1pep1 was fused to the N-terminus, while either Lag3pep1 or Lag3pep2 was incorporated into the loop region of the ferritin monomer, resulting in constructs designated P1L1 and P1L2, respectively (Fig. 2A). For clarity, the parental nanocages are referred to as follows: PD-L1pep1-displaying nanocage as P1; L1 and L2 denote loop-ligated FTH nanocages displaying Lag3pep1 and Lag3pep2, respectively. Both P1L1 and P1L2 ferritin constructs were expressed in *E. coli* and purified to a purity exceeding 98% (Fig. 2B). Three-dimensional modeling of the bispecific nanocages revealed a widespread distribution of PD-L1pep1 across the ferritin surface, whereas the Lag3 binding peptides were observed to form clustered arrangements (Fig. 2C).

DLS analysis revealed that the mean diameters of the P1L1 and P1L2 ferritin nanocages were 26.6 ± 6.1 nm and 20.0 ± 4.9 nm, respectively (Fig. 2D). Zeta potential analysis of ferritin nanocages, including P1L1 and P1L2, yielded −12.1 ± 3.86 mV and −10.6 ± 5.56 mV, respectively, representing a moderate increase compared with that of wFTH (Table 1). TEM analysis confirmed the integrity of the cage architecture, revealing uniform size distributions, indicating that dual-site peptide conjugation did not disrupt proper cage formation (Fig. 2E). Together, these findings confirm that the bispecific PD-L1/Lag3 ferritin constructs, P1L1 and P1L2, are successfully assembled into intact cage structures following expression in *E. coli*.

**Table 1.** Zeta potentials of the ferritin nanocage constructs

wFTH	P1	L2	P1L1	P1L2
−8.36 ± 4.48 mV	−9.12 ± 3.06 mV	−10.9 ± 3.38 mV	−12.1 ± 3.86 mV	−10.6 ± 5.56 mV

### In vitro binding of PD-L1/Lag3 bispecific ferritin nanocages

**Fig. 3.**
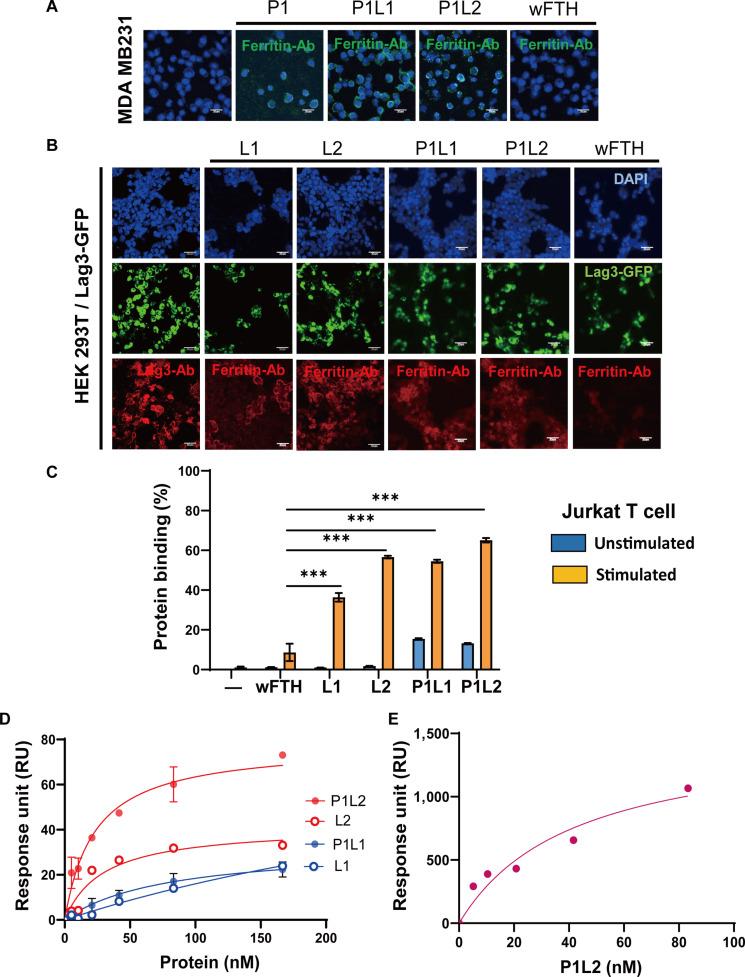
In vitro binding of PD-L1/Lag3 bispecific ferritin nanocages. (A) MDA-MB-231 cells were incubated with P1, P1L1, P1L2, or wFTH at 4 °C for 1 h. Binding interactions were detected using an anti-ferritin antibody (green), and nuclei were counterstained with DAPI (blue). Scale bars: 30 μm. (B) HEK 293T cells expressing Lag3 were incubated with P1L1, P1L2, L1, L2, or wFTH at 4 °C for 1 h. Binding was visualized using an anti-ferritin antibody (red), GFP-Lag3 expression is shown in green, and nuclei were counterstained with DAPI (blue). Scale bars: 30 μm. (C) Jurkat T cells were stimulated with phorbol 12-myristate 13-acetate (PMA), ionomycin, and chloroquine to express Lag3 followed by incubation with P1L1, P1L2, L1, L2, or wFTH. Bound proteins were measured by anti-ferritin antibody with flow cytometric analysis. Statistical comparisons were conducted with Lag3pep displaying nanocages against wFTH (***P < 0.001; one-way analysis of variance [ANOVA]); nonsignificant differences are not shown. (D) SPR analysis of Lag3pep-displaying ferritin constructs (L1, L2, P1L1, and P1L2) against Lag3-coated surface. RU were measured at varying protein concentrations to determine binding affinities (*K*_D_). (E) SPR analysis of P1L2 against PD-L1-coated surface. RU were measured at varying protein concentrations to determine binding affinities (*K*_D_).

We next evaluated the binding interactions of PD-L1/Lag3 bispecific ferritin nanocages, P1L1 and P1L2, with their respective target receptors, PD-L1 and Lag3. Consistent with previous findings, the PD-L1pep mono-displaying ferritin construct, P1, effectively bound to MDA-MB231 human breast cancer cells, which are characterized by high PD-L1 expression [39]. Similarly, the bispecific constructs (P1L1 and P1L2) bound to MDA-MB231 cells, whereas wFTH showed no binding (Fig. 3A). Lag3 binding was firstly assessed using HEK 293T cells transfected with Lag3-GFP because these cells can provide a stable and uniform system with robust Lag3 expression. No basal expression of Lag3 was detected in HEK 293T cells (Fig. S3). Lag3^+^ HEK 293T cells exhibited binding to both Lag3pep mono-displaying ferritin constructs (L1 and L2) and bispecific constructs (P1L1 and P1L2) (Fig. 3B). No binding was observed for wFTH or for any Lag3pep-ferritin constructs on mock-transfected HEK 293T cells (Fig. S4), confirming the specificity of the interaction between Lag3 peptides and the Lag3 receptor. We next evaluated the binding of the ferritin constructs in immune cells expressing endogenous Lag3. Jurkat T cells were stimulated to induce Lag3 expression and subsequently analyzed its binding with ferritin constructs by flow cytometry. The results showed that Lag3pep displaying ferritin constructs (L1, L2, P1L1, or P1L2) exhibited significantly higher binding to Lag3-expressing Jurkat T cells compared with wFTH with P1L2 exhibiting the strongest binding (Fig. 3C).

Binding affinity of the Lag3pep-displaying ferritin constructs (L1, L2, P1L1, and P1L2) to purified Lag3 was further assessed using SPR analysis. The extracellular domain of Lag3 was immobilized and subjected to incubation with different concentrations of ferritin nanocages. Binding responses increased with higher concentrations of Lag3pep ferritin constructs (Fig. 3D and Fig. S5A). The dissociation constants (*K*_D_) for L2 and P1L2 were 29.5 and 22.9 nM, respectively, whereas L1 and P1L1 exhibited *K*_D_ values of 510 and 78.1 nM, respectively (Table 2). These results indicate that Lag3pep2-displaying ferritin constructs (L2 and P1L2) have higher affinity for Lag3 than Lag3pep1-displaying constructs (L1 and P1L1). As expected, wFTH showed no detectable binding to Lag3 (Fig. S5B). Notably, P1L2 exhibited superior binding ability compared with L2, as evidenced by their significantly higher binding responses (*B*_max_), The binding capacity (*B*_max_) can be affected by active accessibility of targeting moieties (Lag3pep2) on the surface of the nanocages. The results might imply that the conformation of 24 Lag3 binding peptides on the surface of P1L2 would be more accessible than L2. Prior studies reported a *K*_D_ of 38 nM for PD-L1pep ferritin (P1) binding to PD-L1 [39]; P1L2 showed a comparable affinity, with a *K*_D_ of 41.89 nM (Fig. 3E and Fig. S5C).

**Table 2.** Binding affinity of Lag3pep-displaying nanocages for the Lag3 receptor

Binding Parameters	L1	L2	P1L1	P1L2
*B*_max_	97.64	41.62	32.83	77.94
*K*_D_ (M)	510.4 × 10^−9^	29.49 × 10^−9^	78.1 × 10^−9^	22.94 × 10^−9^

### Cellular binding of the PD-L1/Lag3 bispecific ferritin nanocages

**Fig. 4.**
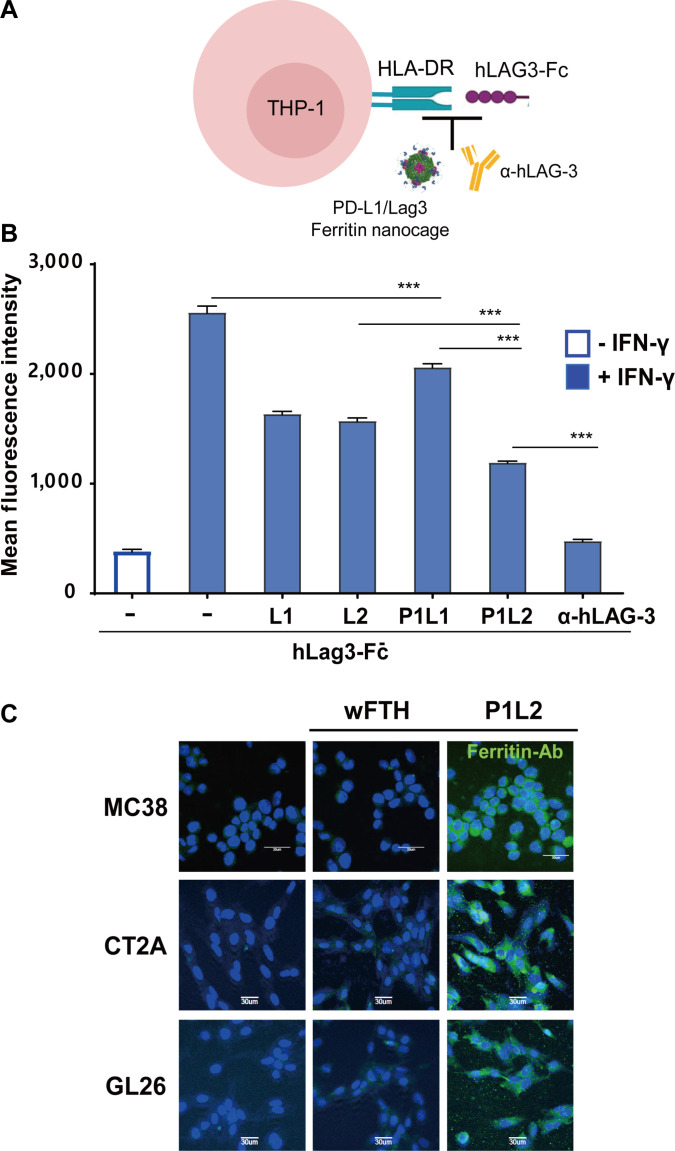
Cellular binding and blocking activity of Lag3pep-displaying and PD-L1/Lag3 bispecific ferritin nanocages. (A) Schematic of the cell-based blocking assay using HLA-DR-expressing THP-1 cells to evaluate the ability of Lag3pep-displaying ferritin nanocages to inhibit the interaction between Lag3 protein and its ligand HLA-DR. (B) Flow cytometry quantification of human recombinant Lag3-Fc binding to HLA-DR-expressing THP-1 cells in the presence of either an anti-human Lag3 blocking antibody (a-hLag3) or Lag3pep-displaying ferritin nanocages. Mean fluorescence intensities are shown. Data are presented as mean ± SD (***P < 0.001; one-way ANOVA); nonsignificant differences are not shown. (C) Mouse colon cancer cells (MC38) and mouse glioma cells (CT-2A and GL26) were incubated with P1L2 or wFTH at 4 °C for 1 h. Binding was detected using an anti-ferritin antibody (green), and nuclei were counterstained with DAPI (blue). Scale bars: 30 μm.

To further evaluate the binding of PD-L1/Lag3 bispecific ferritin nanocages to Lag3, we performed a cell-based blocking assay using HLA-DR-expressing THP-1 cells (Fig. 4A). The optimal concentration of IFN-γ was established by assessing the surface expression levels of HLA-DR (Fig. S6). HLA-DR, a subtype of human MHC-II, is a canonical ligand for Lag3, and their interaction can be inhibited by an anti-Lag3 antibody [43]. Following IFN-γ stimulation of THP-1 cells, Lag3-Fc bound to HLA-DR, and this interaction was effectively blocked by the anti-Lag3 antibody. We then tested whether Lag3pep-displaying ferritin constructs (L1, L2, P1L1, and P1L2) could similarly interfere with Lag3–HLA-DR binding (Fig. 4B). Treatment with either P1L2 or the anti-Lag3 antibody disrupted the interaction, with P1L2 and the anti-Lag3 antibody achieving blocking efficiencies of 63% and 95%, respectively. The lower blocking efficiency of P1L2 reflects the higher binding affinity of the mAb. These results indicate that P1L2 is the most effective ferritin construct for obstructing the functional interaction between Lag3 and its ligand. Based on its specificity and affinity, P1L2 was selected for further development as a PD-L1/Lag3 Bis-ICBN.

To assess in vitro binding of P1L2 to tumor cells, we tested MC38 mouse colon cancer cells and CT-2A and GL26 mouse glioma cells, all of which constitutively express murine PD-L1 [44]. P1L2 exhibited strong binding to all tested murine cancer cell types, whereas wFTH showed no binding, confirming that the observed interactions were mediated by the PD-L1 binding peptides on the P1L2 (Fig. 4C).

### Activation of T-cell responses against tumor cells by the PD-L1/Lag3 Bis-ICBN

**Fig. 5.**
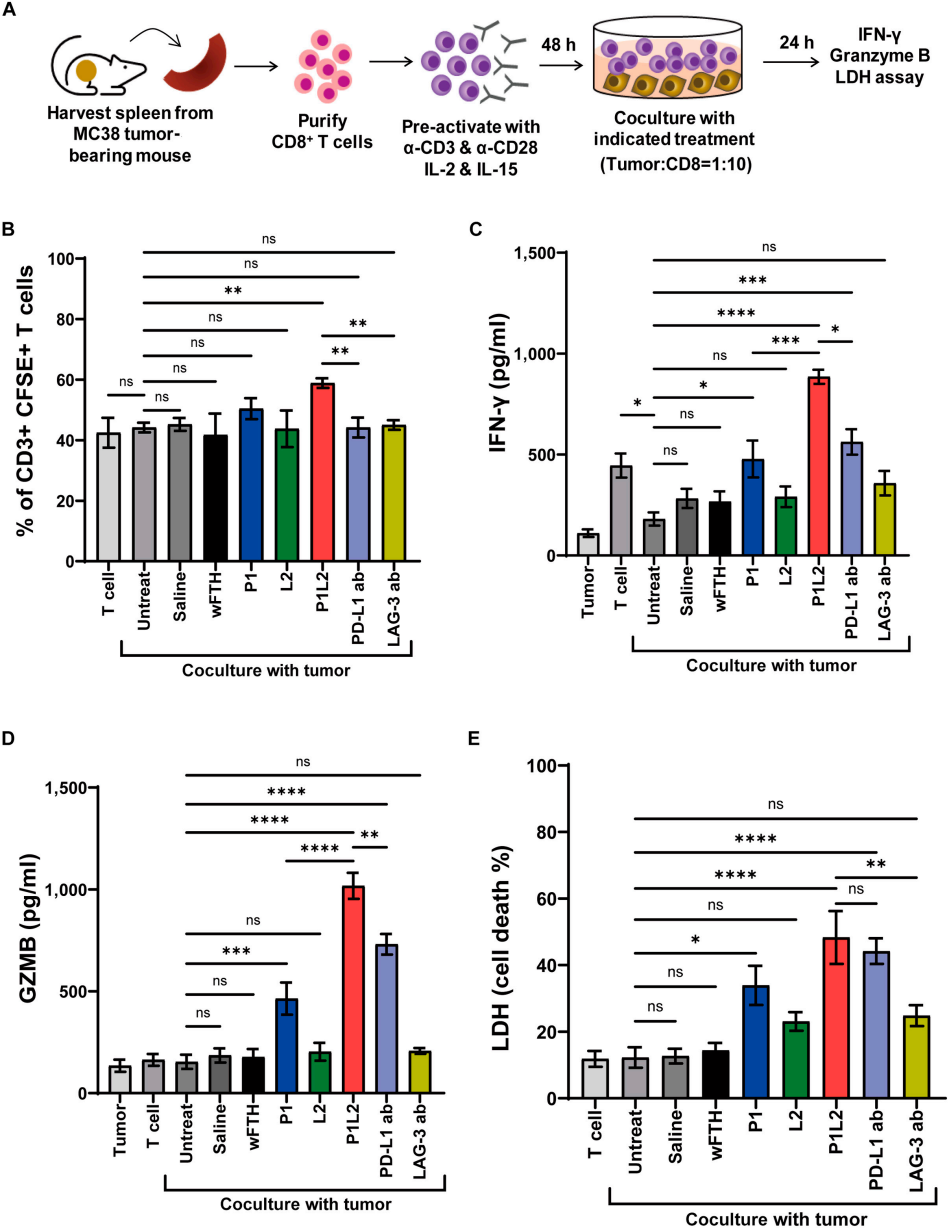
(A) Schematic of the experiment to evaluate the efficacy of P1L2 in enhancing CD8^+^ T-cell activity. CD8^+^ T cells were isolated from the spleens of MC38 tumor-bearing mice, activated, and cocultured with MC38 tumor cells at a T:MC38 ratio of 10:1 for 24 h. Treatments included anti-mouse PD-L1 or Lag3 antibodies (10 μg/ml), ferritin constructs (50 nM), or no treatment. (B) T-cell proliferation was assessed via carboxyfluorescein succinimidyl ester (CFSE) dilution after 24 h of coculture. (C and D) Interferon-gamma (IFN-γ) (C) and Granzyme B (GZMB) (D) levels in the supernatant were quantified by enzyme-linked immunosorbent assay (ELISA). (E) Lactate dehydrogenase (LDH) release was measured as an indicator of tumor cell death. Bar graphs represent mean ± SD. Statistical significance was determined using one-way ANOVA followed by Bonferroni’s test. *P < 0.05, **P < 0.01, ***P < 0.001, ****P < 0.0001, ns; not significant.

To evaluate whether the PD-L1/Lag3 Bis-ICBN, designated P1L2, can enhance T-cell antitumor activity through simultaneous blockade of PD-L1 and Lag3, CD8^+^ T cells were isolated from the spleens of MC38 tumor-bearing mice and subsequently activated (Fig. 5A). The expression of PD-1 and Lag3 on activated CD8^+^ T cells was confirmed (Fig. S7). Coculture experiments were performed with MC38 cells in the presence of P1L2, parental nanocages (P1 or L2), wFTH, or mAbs against PD-L1 or Lag3. Application of P1L2 in the coculture significantly enhanced T-cell proliferation compared to parental nanocages or wFTH, whereas treatment with anti-PD-L1 or anti-Lag3 antibodies did not significantly affect proliferation (Fig. 5B). To further confirm the stimulatory effects of P1L2 on T cells, IFN-γ and Granzyme B secretion were measured across treatment groups. IFN-γ secretion was markedly elevated following P1L2 treatment relative to parental nanocages or antibody treatments (Fig. 5C), and Granzyme B levels were similarly increased in P1L2-treated cells compared to the groups treated with parental nanocages or antibodies (Fig. 5D). These findings suggest that the PD-L1/Lag3 Bis-ICBN, P1L2, robustly activates T-cell immune responses, which may underlie its capacity to inhibit tumor progression in vivo. LDH assays demonstrated a significant enhancement of tumor cell death in cultures treated with P1, P1L2, or anti-PD-L1 antibodies (Fig. 5E). Microscopic examination confirmed tumor cell death in these groups, whereas cells treated with wFTH, L2, or anti-Lag3 antibodies remained largely viable (Fig. S8A). Notably, T cells clustered around tumor cells in P1L2-treated cocultures, unlike other treatment groups (Fig. S8A and B). To selectively assess tumor cell killing in the coculture, MC38 cells and CD8^+^ T cells were labeled with distinct cell-tracking dyes before coculture, and tumor cell survival was quantified by measuring fluorescence from the labeled MC38 cells (Fig. S8C). Treatment with P1, P1L2, or anti–PD-L1 antibodies produced a significant increase in tumor cell death, consistent with LDH assays and microscopic observations. Moreover, tumor cells remained viable even at high concentrations of P1L2 alone, indicating that the observed cytotoxicity was mediated by activated T cells rather than direct effects of the nanocage (Fig. S9).

**Fig. 6.**
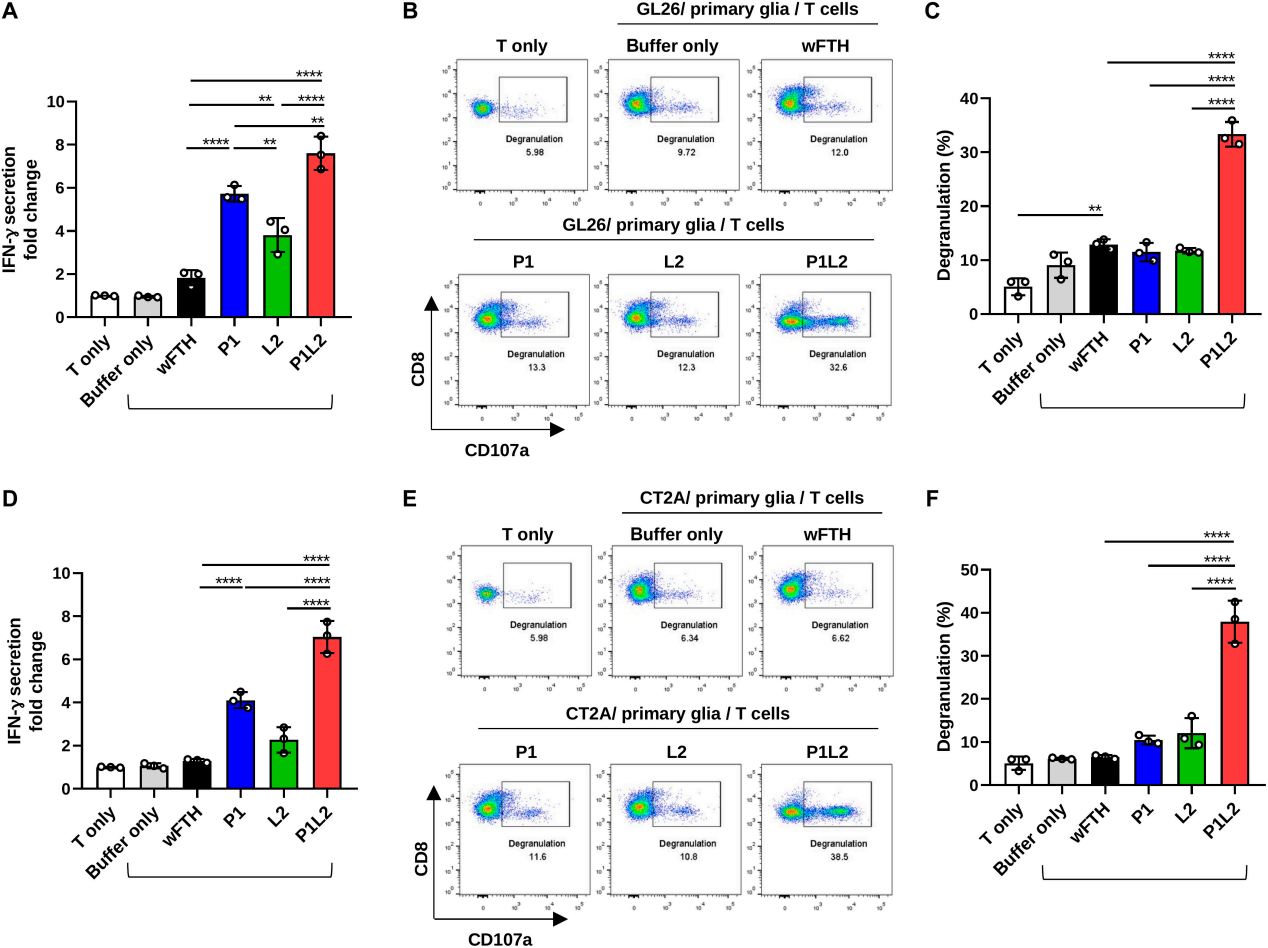
Enhancement of CD8^+^ T-cell responses by P1L2. CD8^+^ T cells were cocultured for 48 h with glioma cells (GL26 or CT2A) in the presence of glial cells and treated with wFTH, P1, L2, P1L2 (25 nM), or vehicle buffer (equal volume). CD8^+^ T-cell functions were assessed by measuring IFN-γ secretion and CD107a expression. (A) Fold change in IFN-γ secretion after coculture with GL26 cells. (B and C) CD107a expression on CD8^+^ T cells after coculture with GL26 cells. (D) Relative IFN-γ levels after coculture with CT2A cells. (E and F) Percentages of CD107a^+^ CD8^+^ T cells after coculture with CT2A cells. Statistical analysis was performed using one-way ANOVA followed by Tukey’s multiple comparisons test. Pairwise comparisons were conducted across all groups; statistical significance between treated groups and controls (T only or buffer only) is not displayed. **P < 0.01, ****P < 0.0001; nonsignificant differences are not shown.

Innate immune cells interact with T cells and promote their activation, leading to the production of immune mediators such as IFN-γ. As brain-resident glial cells function as the primary innate immune effector cells in the brain TME [45], we further investigated whether PD-L1/Lag3 Bis-ICBN, P1L2, enhances T-cell responses against glioma cells in the presence of glial cells in vitro. CD8^+^ T-cell functionality was evaluated by measuring IFN-γ production and degranulation, assessed by measuring surface CD107a expression, in cocultures with GL26 or CT2A glioma cells alongside glial cells (Fig. S10A). During coculture, cells were treated with P1L2 or control nanocages, including wFTH, P1, or L2. In the GL26 system, P1L2 treatment significantly increased IFN-γ secretion from CD8^+^ T to treatment with either P1 or L2 alone (Fig. 6A). Notably, P1L2 induced a marked increase in CD8^+^ T-cell degranulation relative to other nanocage treatments, demonstrating its superior capacity to enhance T-cell activity (Fig. 6B and C and Fig. S10B). Similar trends were observed in the CT2A coculture system, with P1L2 treatment resulting in enhanced T-cell functions compared to other nanocage groups (Fig. 6D to F). Collectively, these findings substantiate the premise that PD-L1/Lag3 Bis-ICBN can effectively stimulate T-cell antitumor immunity and potentially inhibit tumor growth in vivo.

### In vivo tumor homing of the PD-L1/Lag3 Bis-ICBN

**Fig. 7.**
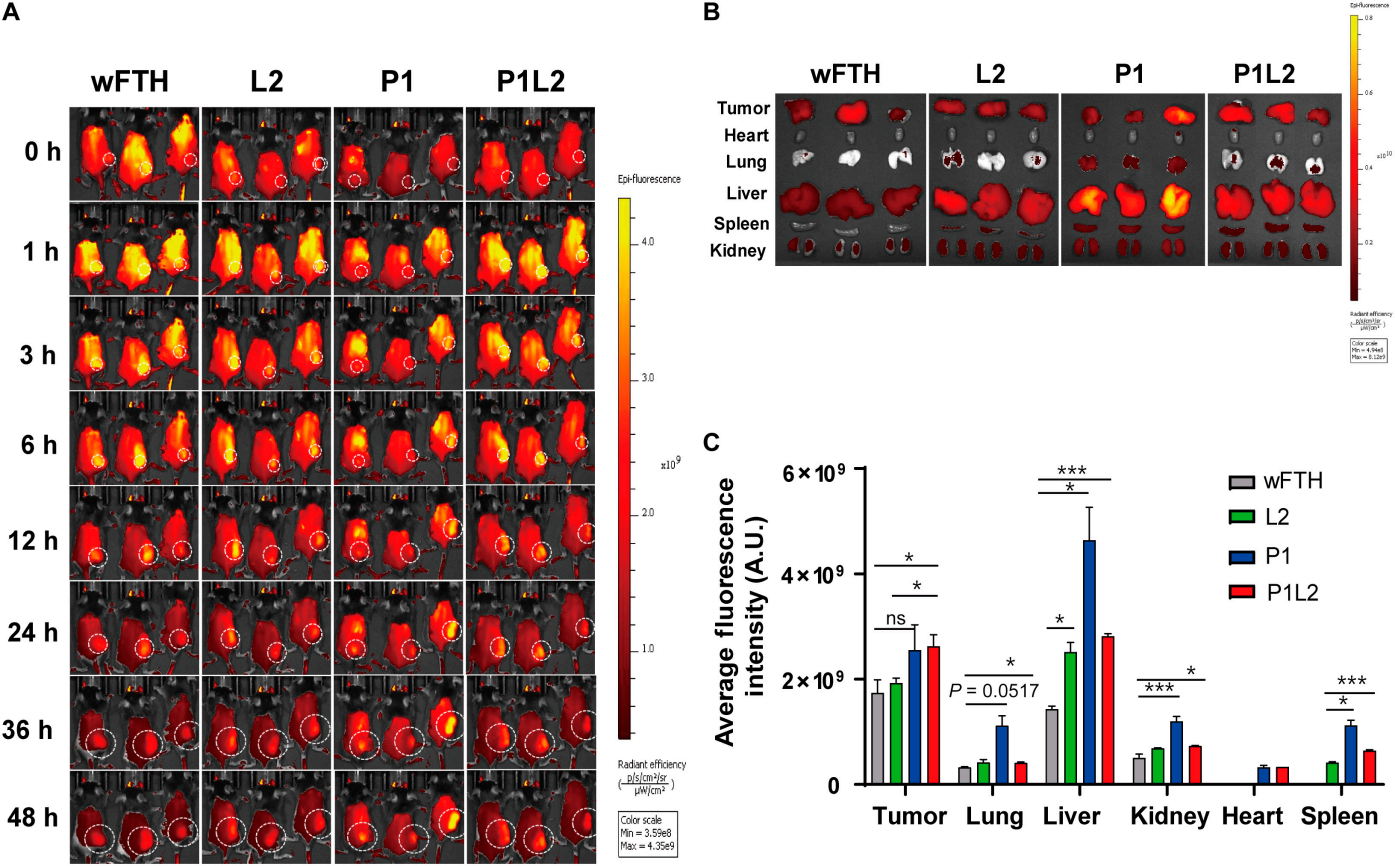
Biodistribution of P1L2 in the MC38 allograft mouse model. (A) Whole-body fluorescence imaging showing the biodistribution of P1L2, P1, L2, and wild-type ferritin (FTH) following intravenous injection of Flamma 675-labeled ferritin constructs using the IVIS imaging system. (B) Ex vivo biodistribution analysis at 48 h postinjection. (C) Quantification of the average fluorescence intensity in each organ at 48 h postinjection. Data are presented as mean ± SE (*P < 0.1, ***P < 0.001, ns; not significant; *t* test).

To assess tumor targeting efficacy, an allograft model was developed by implanting MC38 murine colon cancer cells into mice, followed by evaluation of the in vivo tumor-homing capabilities of the PD-L1/Lag3 Bis-ICBN, designated P1L2. Mice bearing MC38 syngeneic tumors received intravenous injections of fluorescence-labeled ferritin constructs—P1, L2, or P1L2—or a control wFTH. Whole-body fluorescence imaging was performed at multiple time points (Fig. 7A). The results indicated that nanocages displaying PD-L1pep (P1 or P1L2) accumulated more efficiently at tumor sites compared to L2 or wFTH. Notably, strong fluorescence signals persisted in tumors of mice injected with PD-L1pep nanocages (P1 or P1L2) for up to 48 h, whereas signals from L2 or wFTH diminished after 12 h. Ex vivo imaging and quantitative fluorescence analysis of excised organs corroborated the enhanced tumor localization of PD-L1 peptide nanocages (P1 and P1L2) (Fig. 7B and C). The limited tumor homing observed with L2 or wFTH was likely due to passive delivery through the EPR effect.

### Antitumor activity of the PD-L1/Lag3 Bis-ICBN

**Fig. 8.**
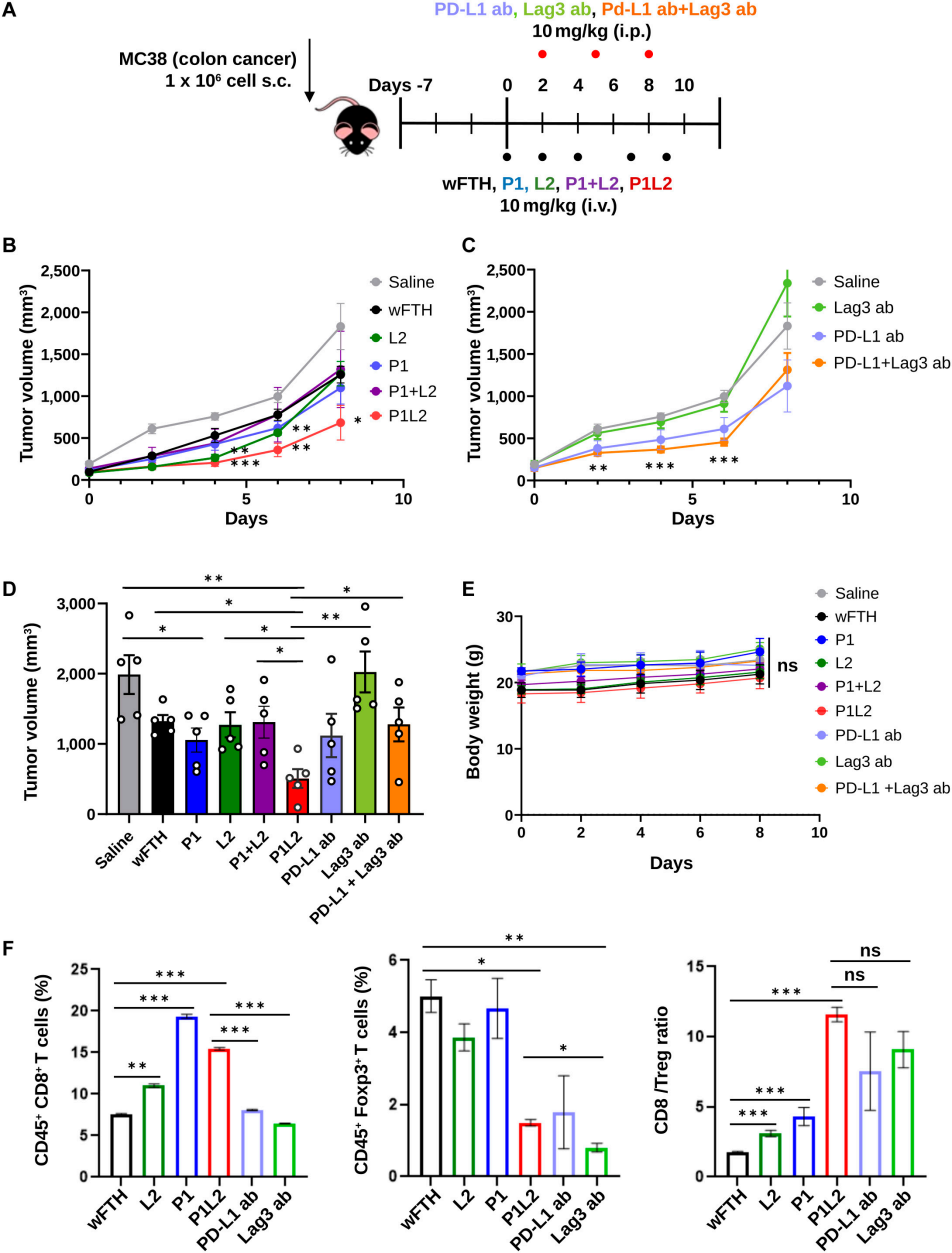
(A) Experimental design for antitumor treatment. MC38 syngeneic colon tumor cells were subcutaneously implanted into mice, and treatment began once tumor volumes reached approximately 50 to 100 mm^3^. P1L2, P1, L2, P1 + L2, or wFTH were administered intravenously 3 times weekly, while anti-PD-L1 or anti-Lag3 antibodies were injected intraperitoneally twice weekly. (B and C) Tumor growth curves during treatment. Statistical significance was determined using 2-way ANOVA followed by Dunnett’s multiple comparison test (*P < 0.05, **P < 0.01, ***P < 0.001); nonsignificant differences are not shown. (D) Final tumor volumes at the end of the study, showing significant inhibition with P1L2 (**P < 0.01). Data are presented as mean ± SE (*P < 0.05, **P < 0.01; *t* test). (E) Body weight changes (ns, not significant; 2-way ANOVA followed by Dunnett’s multiple comparison test). (F) Flow cytometry analysis of CD8^+^, Treg (FoxP^3+^), and ratio of CD8^+^/Treg cells in tumor tissues (*n* = 5 per group). Data are shown as mean ± SE (*P < 0.05, **P < 0.01, ***P < 0.001; ns, not significant; one-way ANOVA).

To evaluate whether the tumor-targeted PD-L1/Lag3 Bis-ICBN, P1L2, effectively suppresses tumor growth, an MC38 allograft mouse model was prepared. Tumor-bearing mice received intravenous injections of P1L2, P1, L2, P1 + L2, or wFTH 3 times weekly for a total of 4 doses. For comparison, mAbs against PD-L1 or Lag3, either alone or in combination, were administered via 3 intraperitoneal injections (Fig. 8A). Treatment with P1L2 demonstrated pronounced antitumor effects, significantly reducing tumor growth relative to the parental P1 or L2 treatments, whether given individually or in combination (Fig. 8B). Administration of PD-L1 and Lag3 antibodies, either alone or in combination, also inhibited tumor growth (Fig. 8C). Notably, the antitumor efficacy of P1L2 surpassed that observed with the combined anti-PD-L1 and Lag3 antibody therapy (Fig. 8D). To further evaluate the in vivo antitumor efficacy, additional histological analyses were performed using H&E staining and TUNEL assays on tumor tissues (Fig. S11). Notably, the tumors from the P1L2-injected mice showed a markedly higher number of TUNEL-positive cells, indicating potently increased apoptotic signals in tumors than the parental P1- or L2-injected mice, whether given individually or in combination. Substantial TUNEL-positive cells were also observed in tumors from the combined anti-PD-L1 and Lag3 antibody treatment. Consistent with these findings, H&E staining demonstrated substantial histological alterations in tumors from the P1L2-treated group, including reduced tumor cellularity and extensive tissue disruption, indicating significant tumor damage.

Throughout the treatment period, no significant differences in body weight were detected among the experimental groups (Fig. 8E), and no adverse effects were observed in hematological parameters, including blood cell counts, or in liver and kidney function when compared to healthy controls (Figs. S12 and S13).

To further elucidate immune responses, tumor-infiltrating leukocytes were examined by gating on CD45^+^ cells. Treatment with both P1 and P1L2 resulted in an increased proportion of transferred CD8^+^ T cells relative to other experimental groups. Conversely, the frequency of CD4^+^FoxP3^+^ regulatory T (Treg) cells was diminished in mice receiving P1L2 or antibody treatments. As a result, the CD8^+^/Treg ratio, a critical indicator of antitumor immunity, was significantly elevated in the P1L2 and anti-PD-L1 groups compared to the P1, L2, or wFTH. These results indicate that the PD-L1/Lag3 Bis-ICBN, P1L2, effectively potentiates antitumor immune responses in colon cancer (Fig. 8F).

## Discussion

Therapeutic strategies that coblockade Lag3 and PD-1/PD-L1 have proven effective in restoring the function of exhausted T cells, thereby enhancing antitumor responses in both preclinical studies and clinical settings [9]. Within the TME, the simultaneous inhibition of PD-1/PD-L1 and Lag3 pathways targets distinct but complementary mechanisms of T-cell suppression. This bispecific blockade not only optimizes immune checkpoint inhibition but also reduces systemic toxicity compared to single-agent therapies. In line with this rationale, we developed a PD-L1/Lag3 Bis-ICBN based on the inherent advantages of ferritin nanocages as antibody-alternative scaffolds.

By simultaneously displaying 24 copies each of PD-L1 binding and Lag3 binding peptides on the surface of human ferritin, we engineered 2 bispecific Bis-ICBN candidates. Among these, the optimized Bis-ICBN, designated P1L2, demonstrated stronger binding affinity to Lag3 and effectively inhibited its interaction with the ligand HLA-DR. Functional analyses revealed that P1L2 significantly promoted T-cell proliferation and cytokine production in cocultures of CD8^+^ T cells with MC38 murine colon cancer cells, surpassing the efficacy of its parental nanocages as well as mAbs targeting PD-L1 or Lag3. Notably, P1L2 was able to restore T-cell functionality within a complex triple coculture system designed to replicate the immunosuppressive TME characteristic of glioblastoma. In cocultures involving CT2A or GL26 glioma cells, glial cells, and CD8^+^ T cells, treatment with P1L2 significantly elevated IFN-γ secretion and T-cell degranulation relative to controls (wFTH or parental nanocages). These results underscore the capacity of P1L2 to potentiate CD8^+^ T cell activity within the brain TME, where immunosuppression is a major therapeutic hurdle.

To evaluate the in vivo immunotherapeutic efficacy of P1L2, its tumor-targeting capability and antitumor activity were tested in a mouse colon cancer model. Although PD-L1 and Lag3 binding peptides were originally identified as ligands for human PD-L1 and Lag3, they also exhibit binding affinity for their murine counterparts [39]. Following intravenous administration in mice bearing MC38 syngeneic colon tumors, P1L2 specifically targeted PD-L1 on tumor cells and remained localized at the tumor site for up to 48 h. The ex vivo biodistribution analysis demonstrated that P1 and P1L2 exhibited greater accumulation in the lung and other organs compared to wFTH. This phenomenon is likely attributable to the elevated levels of circulating IFN-γ, which up-regulated PD-L1 expression on lung myeloid cells as well as on antigen-presenting cells (APCs) within other organs, as observed in a syngeneic colon carcinoma model [46].

P1L2 achieved stronger inhibition of tumor growth than the parent nanocage, either alone or in combination. Notably, P1L2 demonstrated greater antitumor efficacy than the combined treatment with anti-PD-L1 and anti-Lag3 mAbs by enhancing antitumor immunity of CD8^+^ T cells in TME.

Although P1L2 activated CD8^+^ T cells more effectively than P1 in in vitro coculture assays (Figs. 5 and 6), fewer CD8^+^ T cells were detected in tumors from P1L2-treated mice than from P1-treated mice (Fig. 8F). This discrepancy likely reflects the influence of multiple TME factors on in vivo CD8^+^ T-cell recruitment and survival—including T-cell-recruiting chemokines, intratumoral antigen presentation, vascular and extracellular matrix barriers, and the abundance of immunosuppressive cell populations [47]. On the other hand, tumor-infiltrating Tregs were significantly reduced in P1L2-treated mice compared with P1- or L2-treated groups, suggesting that bispecific blockade more effectively decreases intratumoral Treg numbers or suppressive function than monotherapy. P1L2 simultaneously targets 2 pathways critical to Treg biology—PD-1/PD-L1 and Lag3. Tumor-infiltrating Tregs often express high Lag3 levels that support their suppressive function, stability, and proliferation [13]. By blocking Lag3, P1L2 may impair Treg survival and proliferation within the TME. Additionally, PD-1/PD-L1 interactions between APCs/tumor cells and naïve CD4^+^ T cells promote peripheral Treg induction and maintenance; PD-L1 blockade can therefore limit de novo Treg generation in tumor-draining lymph nodes and tumors. The bispecific engagement of APCs/tumor cells with T cells by P1L2 may potentiate these effects by more robustly activating effector T cells and APCs relative to P1 alone. Although the precise mechanisms require further study, the enhanced antitumor efficacy of P1L2 is reflected in the increased CD8^+^ T cell-to-Treg ratio observed in treated tumors.

Given that PD-L1 and Lag3 are expressed on diverse immune cell populations and contribute to immunosuppressive pathways within the intricate TME, it is plausible that P1L2 mediates antitumor immune responses via additional mechanisms. For instance, the concurrent engagement of PD-L1 and Lag3 may facilitate DC activation and promote interactions between tumor cells and T cells, thereby augmenting the function of cytotoxic CD8^+^ T cells [48]. Moreover, PD-L1 is abundantly expressed on TAMs within the colorectal cancer TME, and blockade of PD-L1 with specific antibodies has been shown to attenuate the immunosuppressive activity of TAMs [46]. Consequently, P1L2 may be effective across various TME contexts, although the precise molecular mechanisms underlying its activity warrant further investigation. Additionally, several bispecific antibodies targeting Lag3 and PD-1/PD-L1, including MGD013, FS118, IBI323, and ABL501, are currently undergoing preclinical and early clinical evaluation, demonstrating efficacy comparable or superior to conventional combinations of PD-1/PD-L1 and Lag3 mAbs [48–50]. The therapeutic effectiveness of P1L2 compared to these bispecific antibodies also remains an open question.

Overall, the results suggest that the PD-L1/Lag3 Bis-ICBN, P1L2, is a promising bispecific immune checkpoint nanomedicine for cancer therapy. Furthermore, this bispecific nanocage has the potential to encapsulate small chemotherapeutic agents, which may further enhance its antitumor efficacy across a wide range of solid tumors, highlighting its promise as a next-generation immunotherapeutic nanomedicine.

In this study, we engineered a bispecific immune checkpoint blockade nanocage (Bis-ICBN) presenting 24 peptides targeting PD-L1 and 24 peptides targeting Lag3 on its surface. This PD-L1/Lag3 Bis-ICBN is designed to selectively localize to tumors via specific binding to PD-L1, while concurrently inhibiting the immune checkpoint receptors PD-L1 and Lag3 within the TME, thereby suppressing tumor growth. Furthermore, these bispecific nanocages may facilitate enhanced recruitment of T cells to the tumor site, potentially eliciting a more robust antitumor immune response compared to the combined administration of mAbs directed against PD-L1 and Lag3.

## Data Availability

All raw data are available upon request from the corresponding author.
