# Supplementary material for: PD-L1/Lag3 Bispecific Immune Checkpoint Blocking Nanocage Exhibits Potent Antitumor Activity beyond Dual Blockade of PD-L1 and Lag3
Source: Biomater Res. 2026 May 7;30:0362. doi: 10.34133/bmr.0362 (PMC13150080; doi:10.34133/bmr.0362)
Supplement: Supplementary 1 — Figs. S1 to S13 [file bmr.0362.f1.pdf]

Supplementary Materials

PD-L1/Lag3 bispecific immune checkpoint blocking nanocage exhibits potent antitumor activity beyond dual blockade of PD-L1 and Lag3

‡Seok-Min Lee[1,2,3], *‡Minseong Kim*[1,2,3], *Chanju Lee*[4], *Minah Lee*[1,2,3], *Hee Jung Yoon*[4], *Byungheon Lee*[1,2,3], *Eun Jung Park*[4], *Soyoun Kim*[1,2,3]*

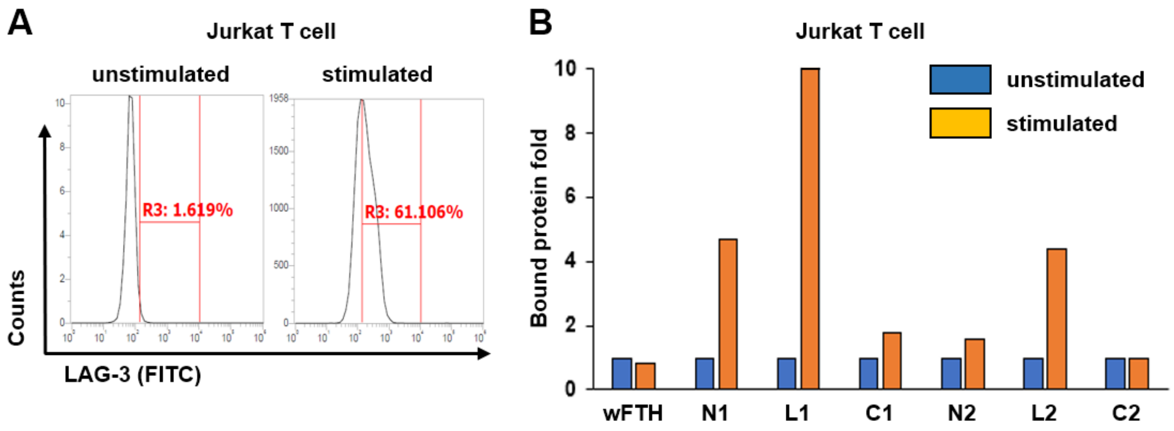

Supplementary Figure 1. Flow cytometric analysis of Lag3pep-presenting ferritin constructs binding to Lag3 on Jurkat T cells. (A) Induction of LAG-3 expression in Jurkat T cells. Jurkat T cells were stimulated with PMA, ionomycin, and chloroquine (CQ) for 48 h to upregulate LAG-3 expression. Representative flow cytometry histograms show LAG-3 expression in unstimulated and stimulated Jurkat T cells. **(B)** Binding of Lag3pep-presenting ferritin constructs to Jurkat T cells. Fluorescence-labeled Lag3pep-displaying ferritin constructs were incubated with either stimulated or unstimulated Jurkat T cells. The binding level was expressed as fold change, calculated as the ratio of mean fluorescence intensity (MFI) between stimulated and unstimulated cells.

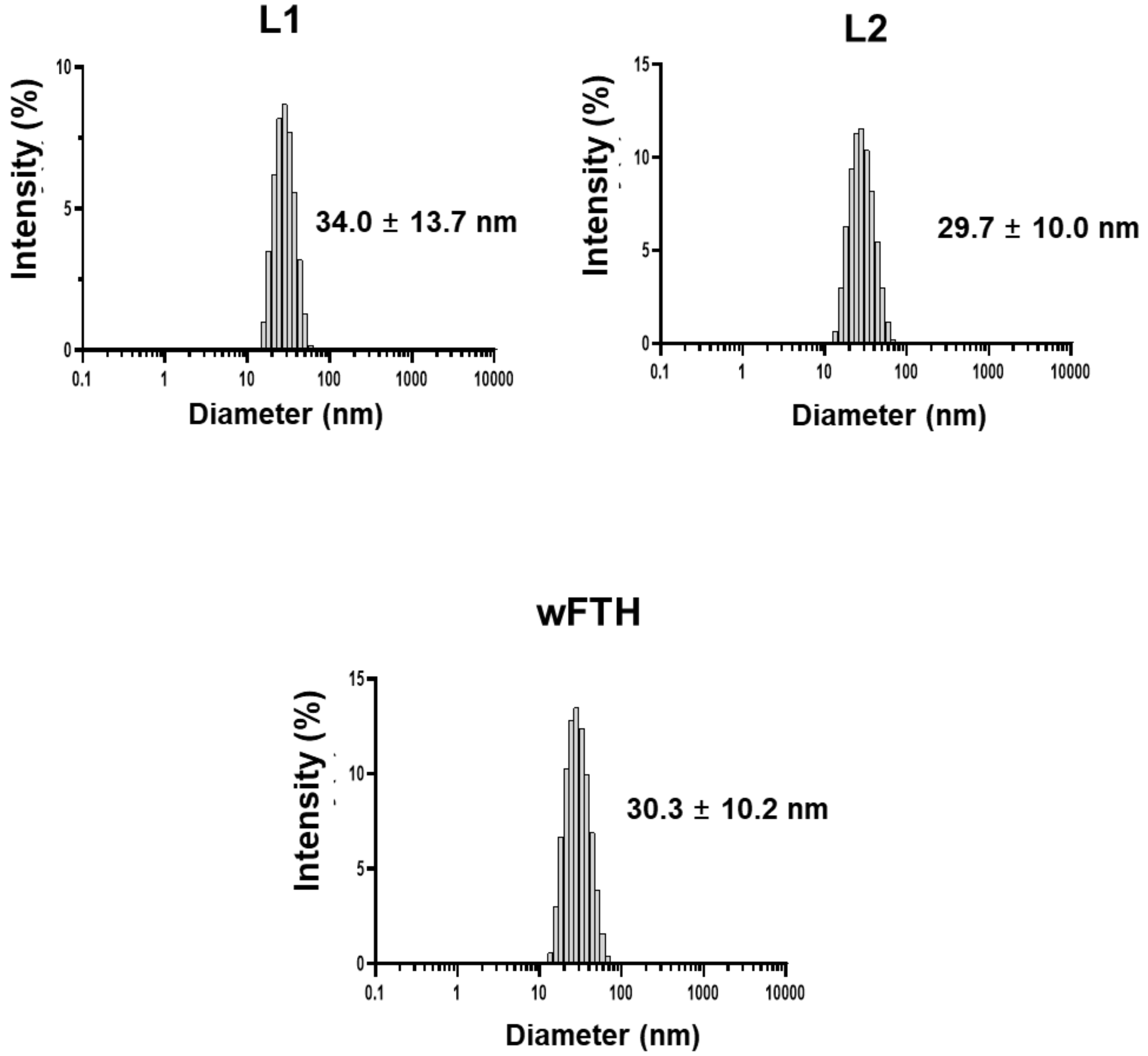

Supplementary Figure 2. DLS analysis showing the size distribution of Lag3pep loop ligated ferritin nanocages (L1 and L2) and wild-type ferritin heavy chain (wFTH).

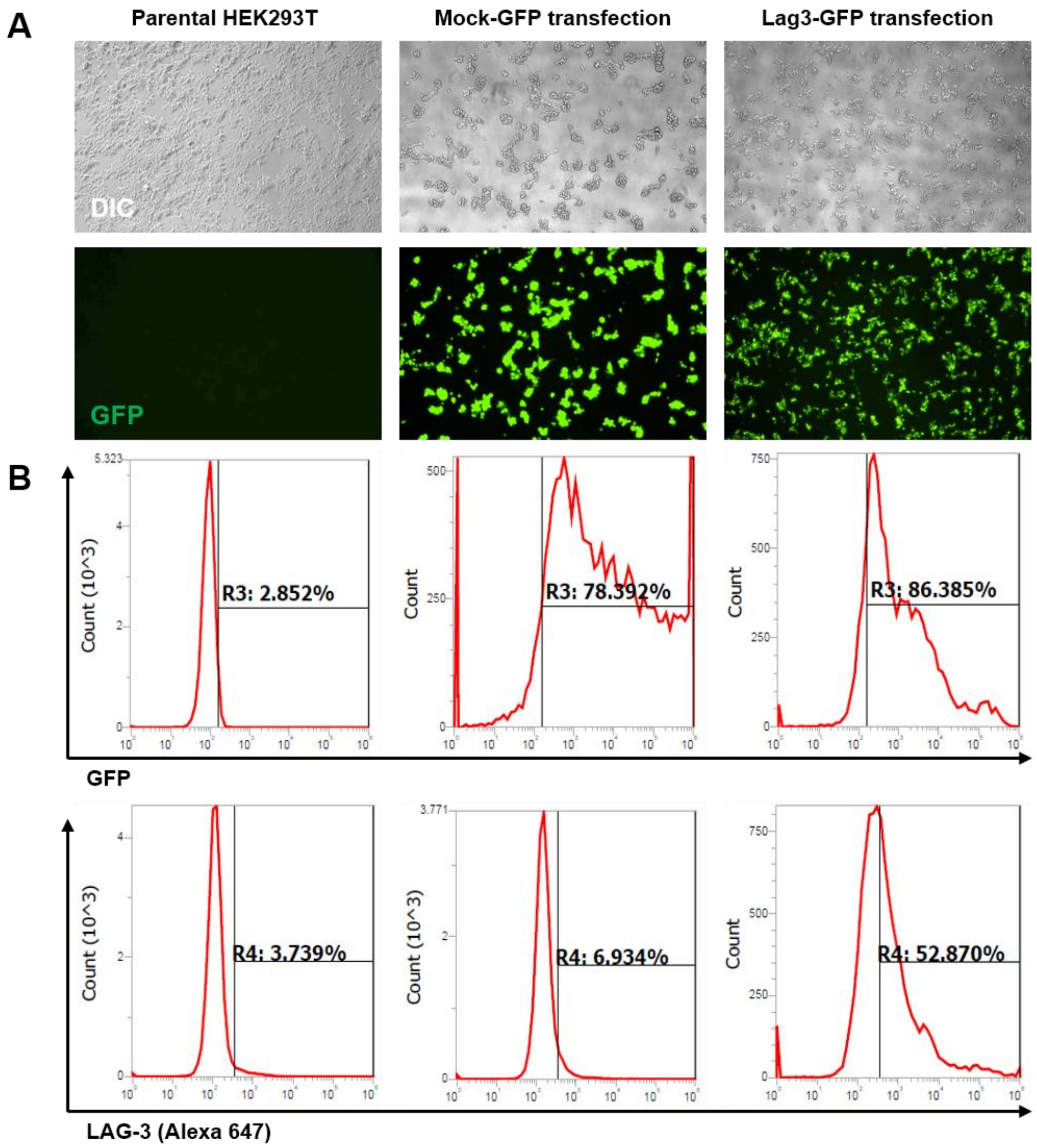

Supplementary Figure 3. Validation of GFP and LAG-3 expression in transfected HEK293T cells. (A) Representative microscopy images of parental HEK293T cells, mock-GFP–transfected HEK293T cells, and Lag3–GFP–transfected HEK293T cells at 48 h post-transfection. Bright-field (DIC) and fluorescence (GFP) images are shown (200×). **(B)** Flow cytometric analysis of GFP and Lag3 expression in HEK293T cells 48 h after transfection. Cells were stained with an anti–Lag3 antibody and analyzed for GFP fluorescence. Representative histograms show the distribution of GFP signal and Lag3 expression in each group.

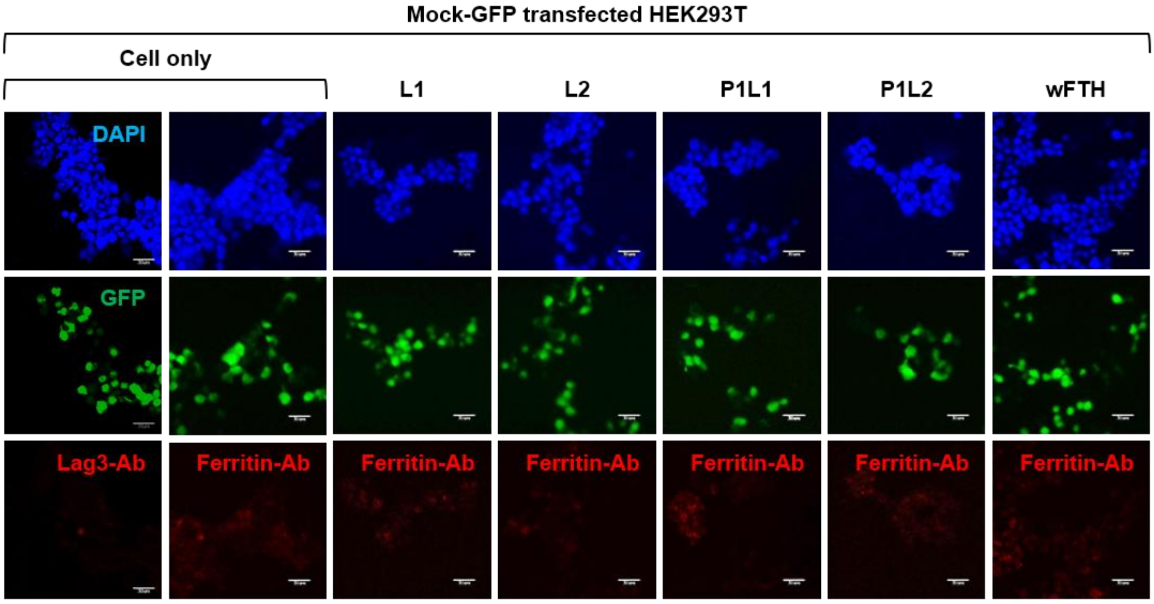

Supplementary Figure 4. *In vitro* cell binding of Ferritin constructs. Neither Lag3pep mono-displaying nanocages (L1, L2) nor PD-L1pep/Lag3pep dual displaying nanocage (P1L1, P1L2) bound to mock-GFP expressing HEK 293T cells. The binding was monitored by anti-ferritin antibody (red). Nuclei were visualized by counterstaining with DAPI (blue). Transiently expressed GFP (green) was shown. Scale bars: 30 μm.

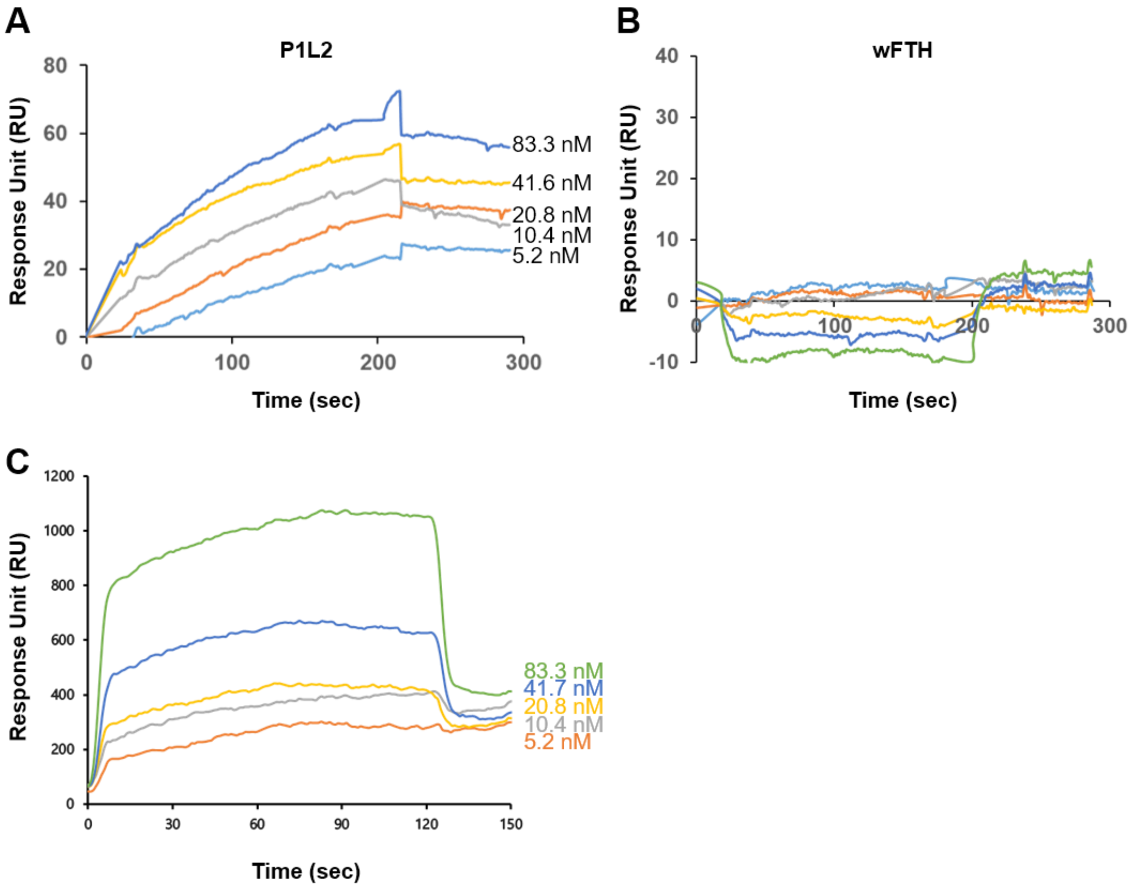

Supplementary Figure 5. ***In vitro*** **binding analysis of P1L2.** Surface Plasmon Resonance (SPR) sensorgram of P1L2 **(A)** or wFTH **(B)** binding to immobilized Lag3. **(C)** RU values of different concentrations of P1L2 were measured against immobilized PD-L1.

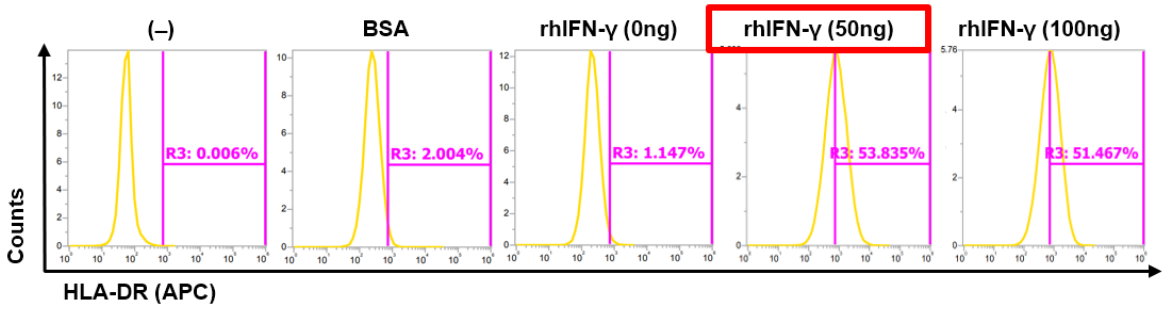

Supplementary Figure 6. HLA-DR expression in THP-1 cells following IFN-γ treatment. THP-1 cells were treated with recombinant human IFN-γ at the indicated concentrations for 48 h. Surface expression of HLA-DR was analyzed by flow cytometry. Representative histograms are shown.

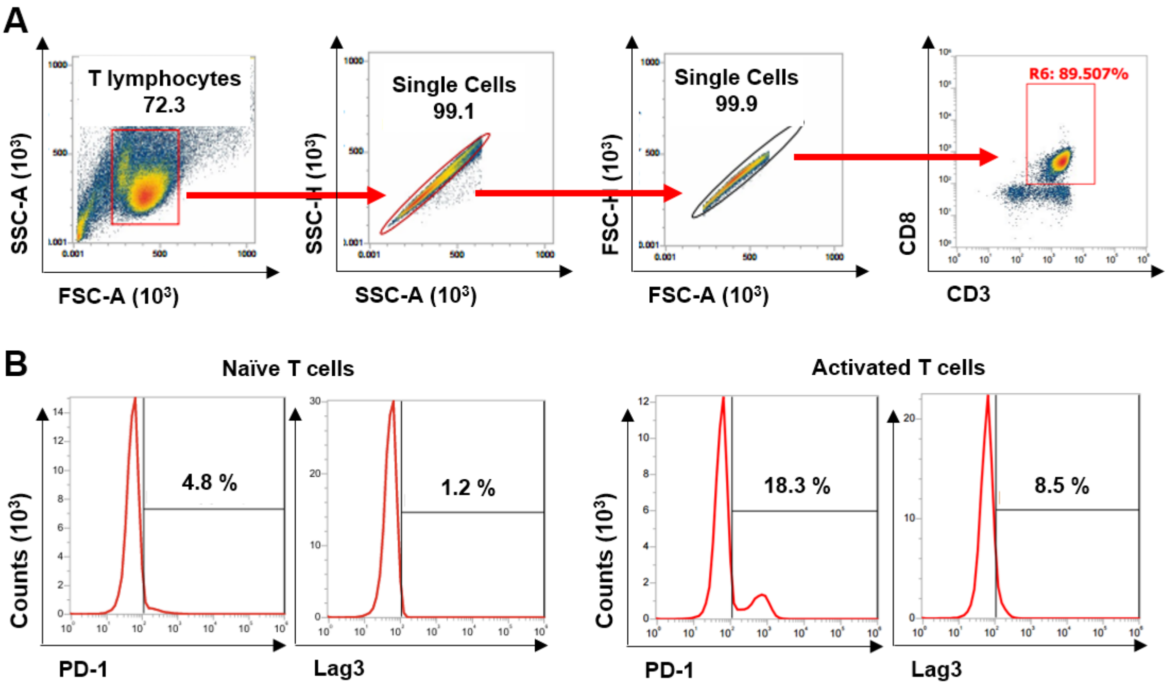

Supplementary Figure 7. Isolation of the activated CD8$^+$ T cells. (A) Gating strategies for activated CD8$^+$ T cells. Lymphocytes were gated based on FSC and SSC, followed by CD3 and CD8 immune cell gate within single cell gate. **(B)** PD-1 and Lag3 expression were measured on naïve vs. activated T cells.

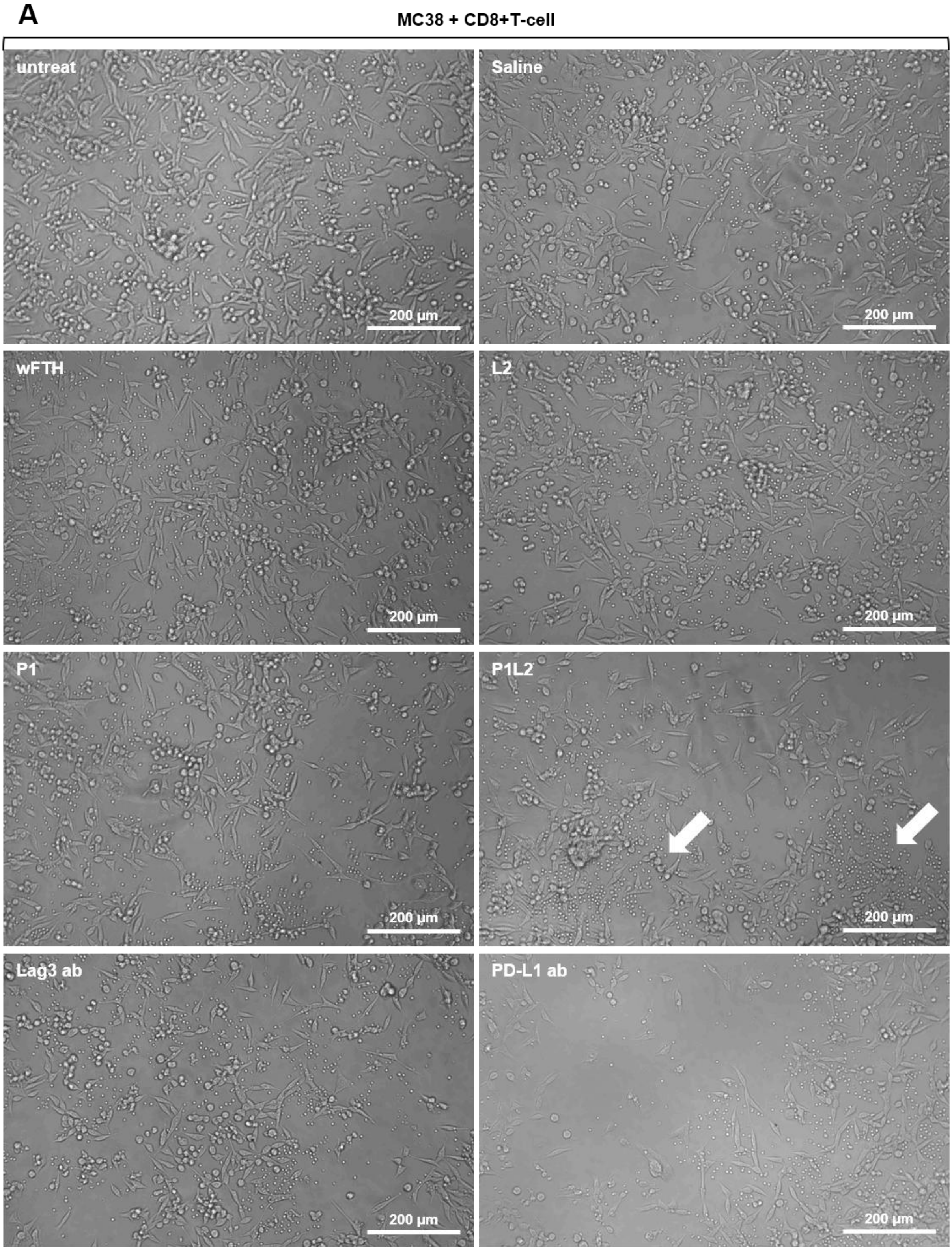
A
MC38 + CD8+T-cell
untreat
Saline
wFTH
L2
P1
P1L2
Lag3 ab
PD-L1 ab
200 µm

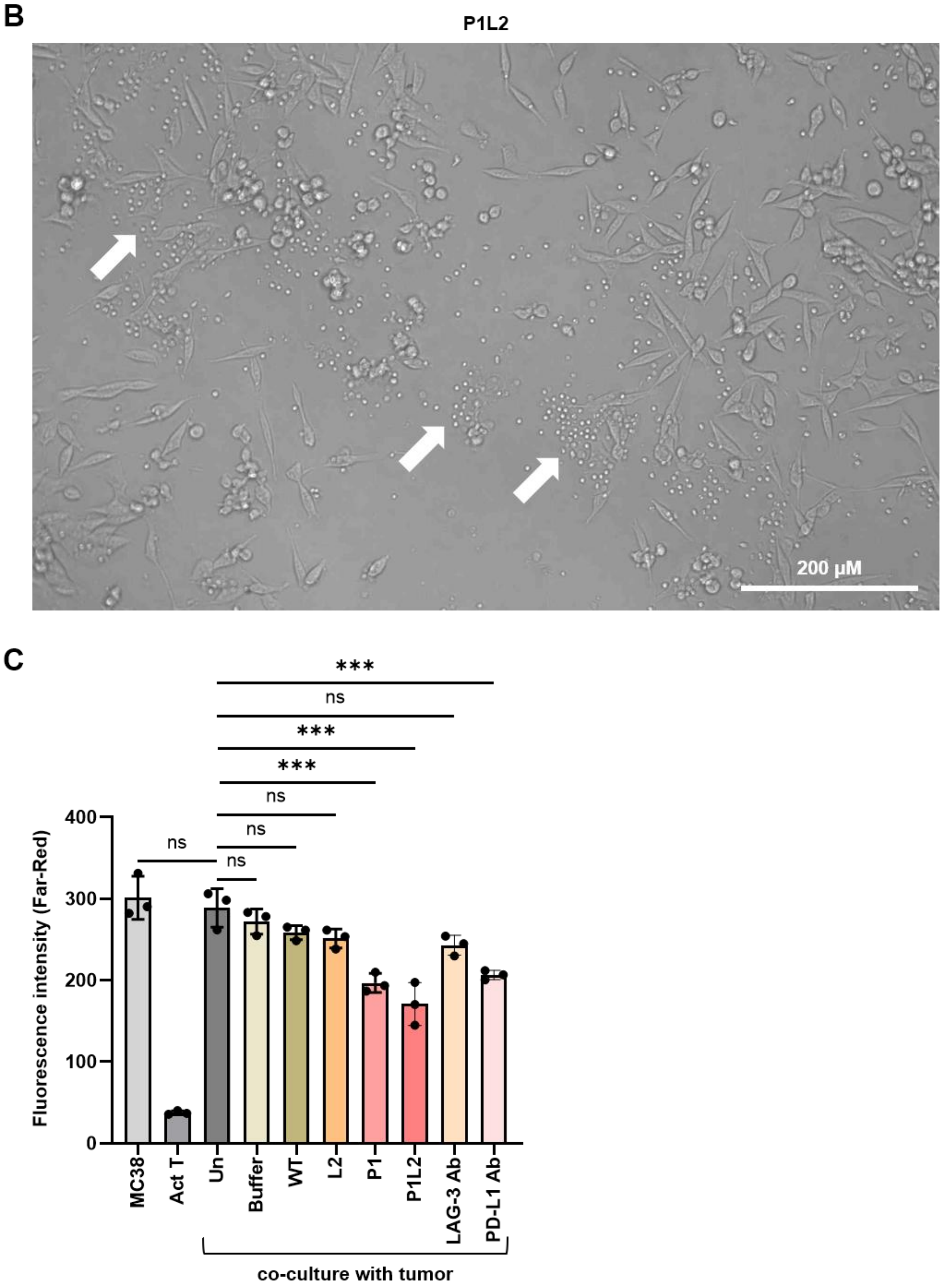

Supplementary Figure 8. Representative images of co-cultured CD8^{+} T cells and MC38 tumor cells (1:10 ratio). (A) The co-cultured cells were treated with mouse antibodies against PD-L1 or Lag3 (10 μg/mL), ferritin constructs (50 nM), or left untreated. The tumor cell population was visibly reduced in the P1L2-, P1-, and anti–PD-L1 antibody–treated groups

under microscopy (200×). **(B)** Magnified image of P1L2-treated co-culture. White arrows (A, B) indicate CD8$^+$ T cell accumulation around tumor cells. **(C)** Quantitative analysis of tumor cell survival. MC38 tumor cells were labeled with a Far-Red cell-tracking dye prior to co-culture. After 24 h of co-culture with CD8$^+$ T cells, the fluorescence intensity derived from labeled MC38 cells was measured using a fluorescence plate reader. The fluorescence signal was used as an indicator of tumor cell viability. Data are presented as relative fluorescence intensity, reflecting the remaining tumor cell population under each treatment condition. Statistical analysis was performed using one-way ANOVA. *$P < 0.05$, **$P < 0.01$, ***$P < 0.001$; ns, not significant.

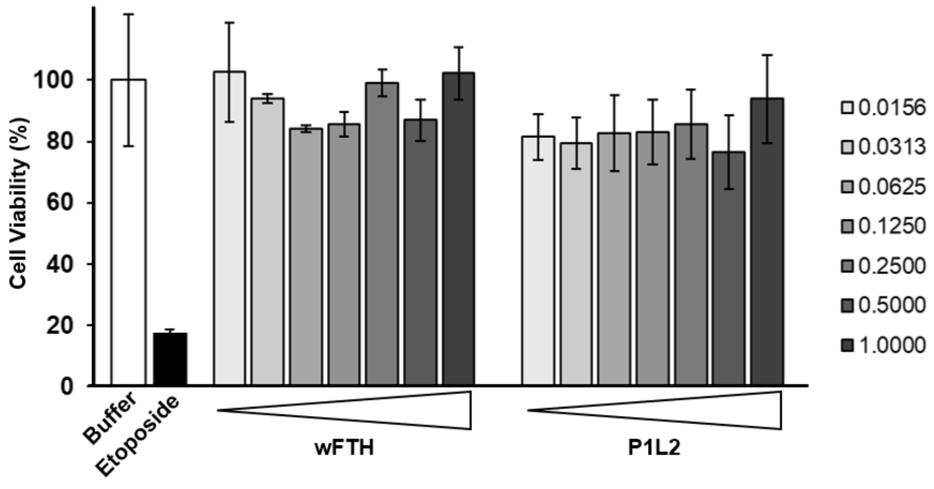

Supplementary Figure 9. The cytotoxicity of P1L2. The MC38 cells (5 × 10^3/well) were seeded on 96-well culture plates, grown in RPMI supplemented with 10% FBS for 24 h, and incubated with P1L2 (0–1 uM) for an additional 48 h. As controls, buffer or etoposide (100 uM) was treated. Cell viability was evaluated using the Cell Counting Kit 8 (CCK8) assay kit (abcam) according to manufacturer's direction. Wild type ferritin (wFTH) was used as controls. Data represent means ± SEM.

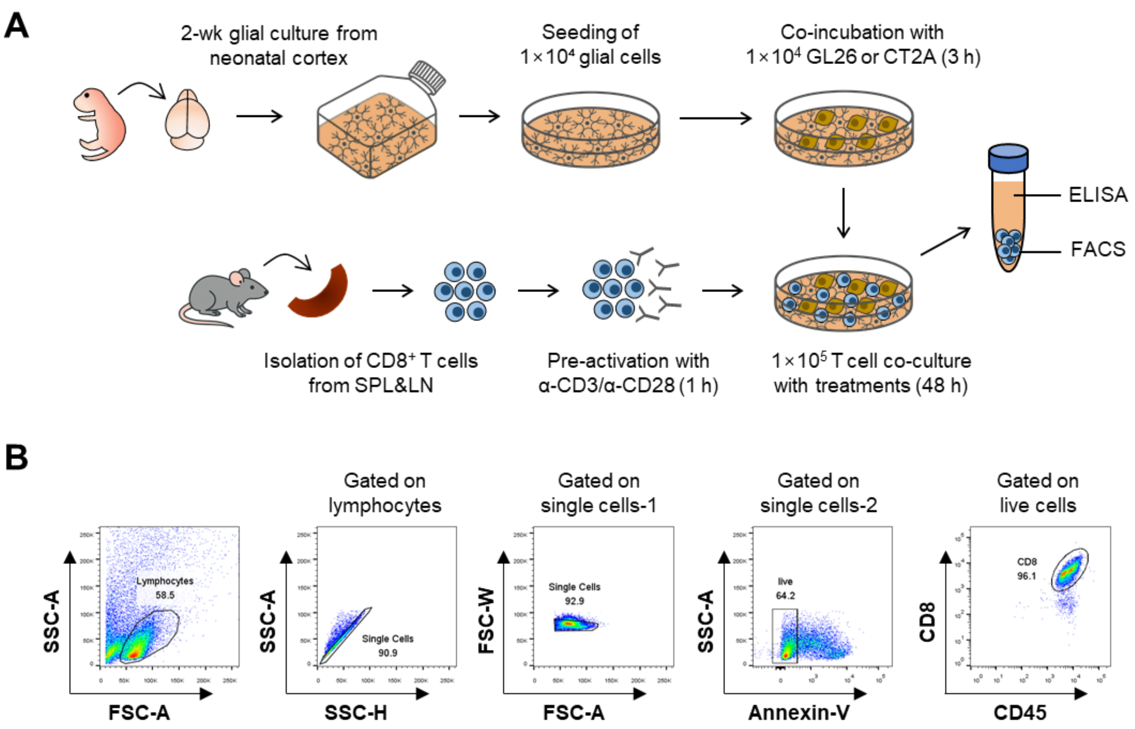

Supplementary Figure 10. Schematic diagram of CD8$^+$ T cell analysis via co-culture with glioma cells and primary glia. (A) CD8$^+$ T cells were isolated from the spleen and lymph nodes of wild-type mice via immunomagnetic negative selection and pre-activated with α-CD3/α-CD28 antibodies for 1 h. They were then co-cultured for 48 h with glioma cells (GL26 or CT2A), which had been seeded onto primary glia prior to co-culture, in the presence of indicated treatments described in Figure 6. Supernatants were collected for IFN-γ ELISA, and cells were analyzed by flow cytometry. **(B)** Representative gating strategy for CD8$^+$ T cells. Lymphocytes were gated based on FSC/SSC, followed by single-cell gating. Dead cells were excluded using Annexin-V$^-$ gating, and CD45$^+$CD8$^+$ live T cells were analyzed.

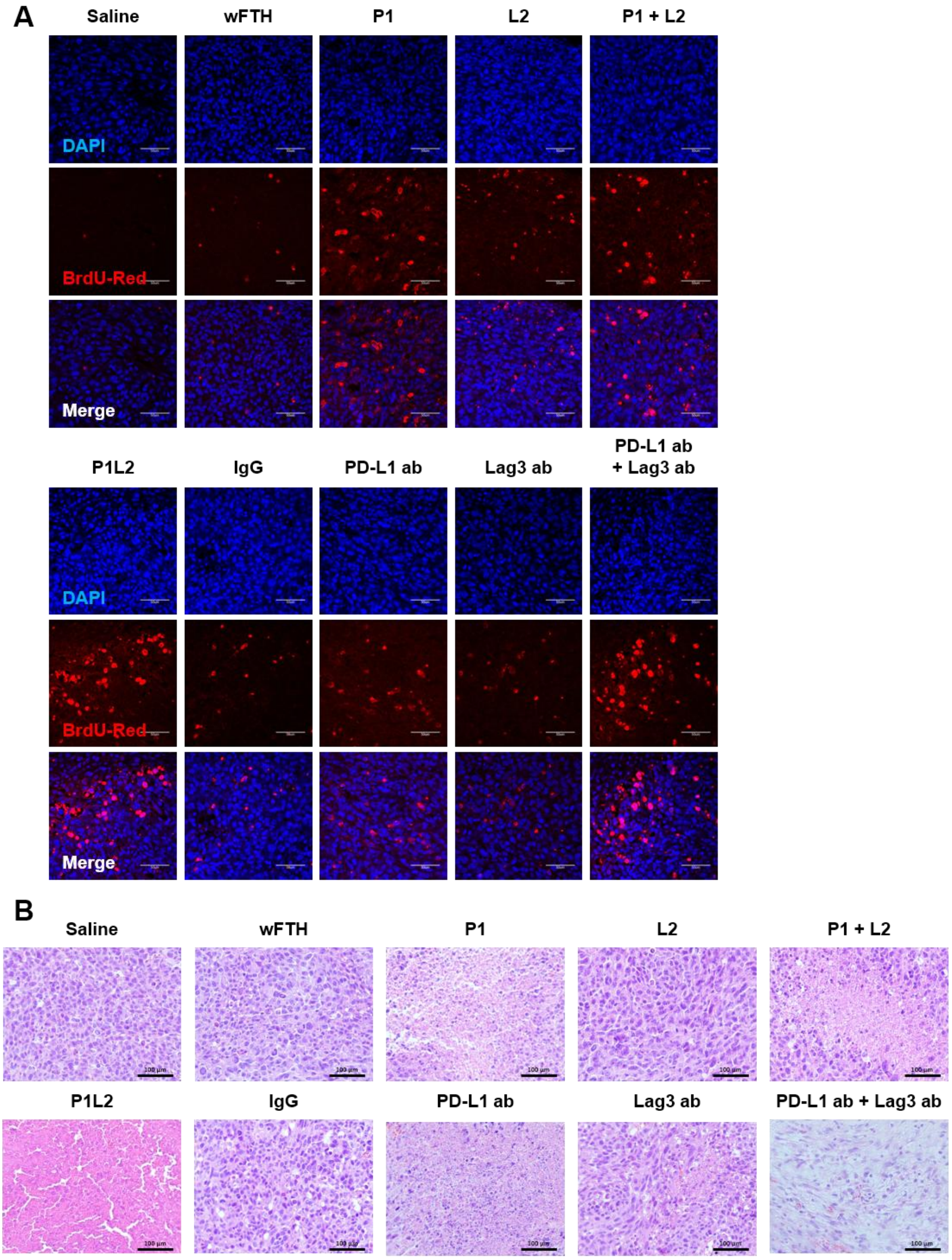

Supplementary Figure 11. Histological evaluation of antitumor efficacy by TUNEL and H&E staining. (A) TUNEL staining of tumor tissues. Paraffin-embedded tumor sections were subjected to TUNEL assay using a BrdU-Red labeling system. Representative fluorescence

images show nuclei (DAPI, blue) and apoptotic cells (BrdU-Red, red). TUNEL-positive cells were observed across treatment groups, with increased signal intensity in selected treatment conditions. Images were acquired at 400× magnification. **(B)** Hematoxylin and eosin (H&E) staining of tumor tissues. Representative images show histological features of tumor tissues under different treatment conditions. Variations in tumor cellularity and tissue architecture were observed among the groups. Images were acquired at 200× magnification.

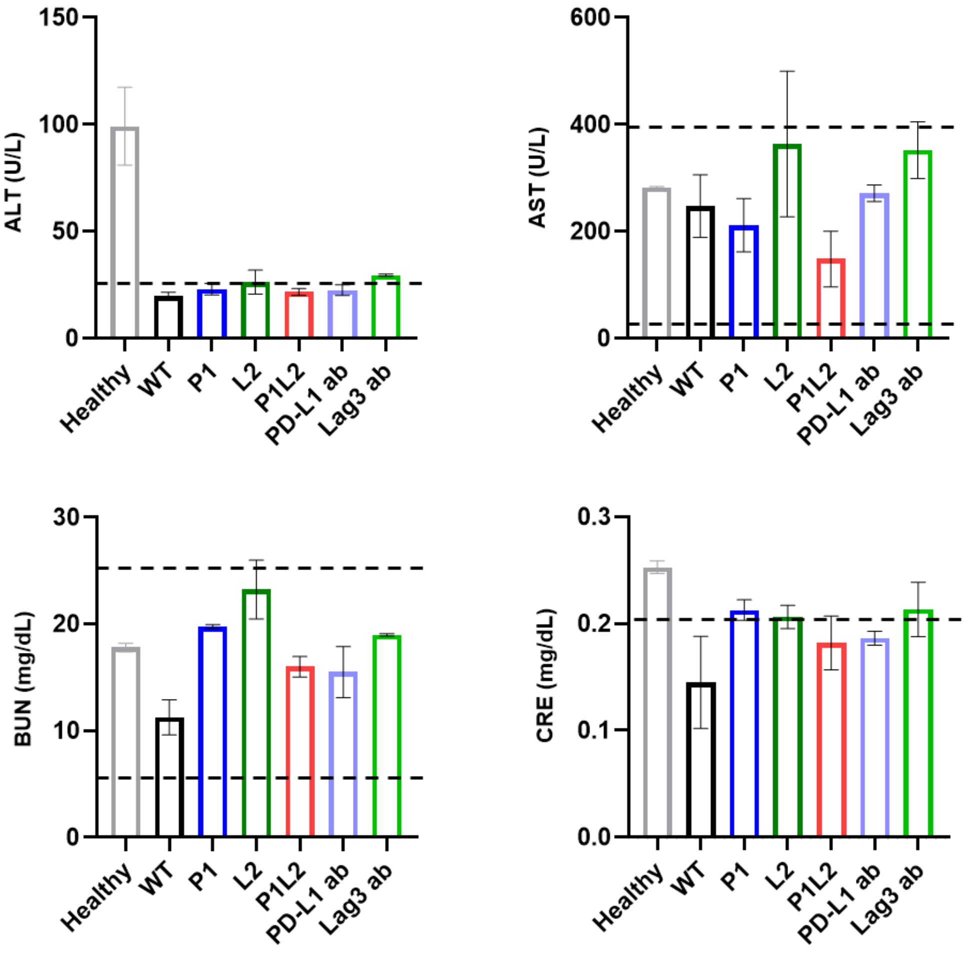

Supplementary Figure 12. Analysis of liver and kidney functions after treatments with ferritin constructs. Levels of ALT (alanine transaminase) and AST (aspartate transaminase) in the blood were measured to test liver function and levels of BUN (blood urea nitrogen) and CRE (creatinine) were measured for kidney function. Healthy mice and treated groups are indicated. Data are mean ± S.E. (n = 3/group). Dotted lines represent normal values of each parameter.

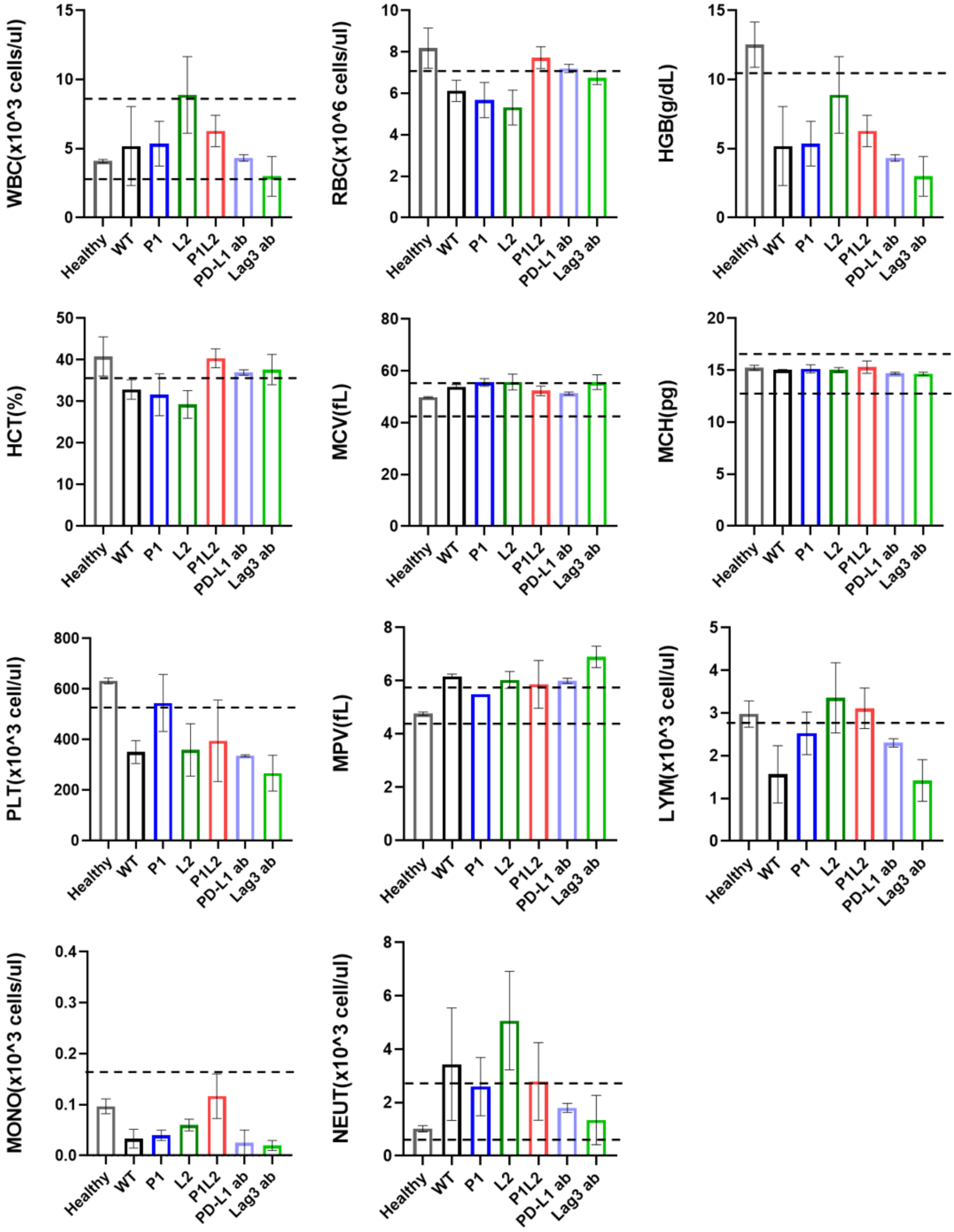

Supplementary Figure 13. Analysis of hematological parameters after treatments with ferritin constructs. The complete blood count test as specified was performed for healthy mice and treated groups. Data are mean ± S.E. (n = 3/group). Dotted lines represent normal values of each parameter.
